# Clusterzymes-driven therapy: ultrasmall Cu_4_ nanoclusters achieve dual-pronged synergistic effects on antioxidant defense and ferroptosis Inhibition for inflammatory osteolysis

**DOI:** 10.1186/s12951-025-04009-2

**Published:** 2026-01-21

**Authors:** Yucheng Wang, Zeyu Han, Guanghua Hao, Han Cheng, Fengrong Dai, Xuzhuo Chen, Bin Shi

**Affiliations:** 1 Department of Oral and Maxillofacial Surgery, The First Affiliated Hospital of Fujian Medical University, 20 Chazhong Road, Fuzhou, 350005 China; 2 School of Stomatology, Fujian Medical University, Fuzhou, 350005 China; 3 Department of Oral Surgery, Shanghai Ninth People’s Hospital, Shanghai Jiao Tong University School of Medicine; College of Stomatology, Shanghai Jiao Tong University; National Center for Stomatology; National Clinical Research Center for Oral Diseases; Shanghai Key Laboratory of Stomatology; Shanghai Research Institute of Stomatology, The Ninth People’s Hospital Affiliated to Shanghai Jiao Tong University, 639 Zhizaoju Road, Shanghai, 200011 China; 4 Department of operating room, Linyi Central Hospital, Linyi, 276400 China; 5 School of Physical Science and Technology, Shanghai Tech University, Shanghai, 201210 China; 6 State Key Laboratory of Structural Chemistry, Fujian Institute of Research on the Structure of Matter, Chinese Academy of Sciences, Fuzhou, 350005 China

**Keywords:** Nanoclusters, Inflammatory osteolysis, Osteoclast, ROS, Nrf2 pathway, Ferroptosis

## Abstract

Inflammatory osteolysis represents a critical complication following orthopedic interventions such as total joint replacement, primarily triggered by persistent inflammatory responses induced by prosthetic wear debris or bacterial components like lipopolysaccharides (LPS). Inflammatory osteolysis, a severe complication of orthopedic interventions like total joint replacement, is driven by prosthetic wear debris or lipopolysaccharides (LPS)-induced persistent inflammation and osteoclast activation. Current therapeutic strategies are limited by significant side effects and their inability to simultaneously halt the synergistic pathological processes of inflammation and osteoclast activation, highlighting an urgent need for novel therapeutic approaches. In this study, we synthesized ultrasmall Cu₄ nanoclusters with potent superoxide dismutase (SOD)-and catalase (CAT)-mimetic activities, enabling efficient reactive oxygen species (ROS) scavenging (80.43% ^•^O_2_^−^ and 93.17% H₂O₂ clearance at 200 µg/mL). we successfully synthesized ultrasmall Cu₄ clusters, which exhibit remarkable enzyme-mimetic activities (superoxide dismutase and catalase-like) and potent reactive oxygen species (ROS)-scavenging capabilities. These clusters specifically target mitochondria, effectively scavenging excessive ROS to mitigate oxidative stress. Furthermore, Cu₄ clusters activate the nuclear factor erythroid 2-related factor 2 (Nrf2) signaling pathway, inhibit the activation of key inflammatory pathways such as nuclear factor-κB (NF-κB), regulate iron homeostasis (Ferro-orange staining showed that the positive cell ratio in the lps group was as high as 54.1%, while it dropped to 9.96% in the 20 µg/mL Cu₄ clusters group) and lipid peroxidation to block ferroptosis, and reduce osteoclast formation. In LPS-induced calvarial osteolysis mice, Cu₄ clusters significantly alleviated bone resorption, restoring bone volume/tissue volume (BV/TV) by 57.6% (91.7% of control group) and reducing osteoclast number to 36.4% of the LPS group.Collectively, these actions result in significant alleviation of inflammation and bone resorption. This study highlights Cu₄ clusters as a promising therapeutic agent for inflammatory osteolysis, with substantial potential for clinical translation.

**Graphical Abstract:**

**Figure:**
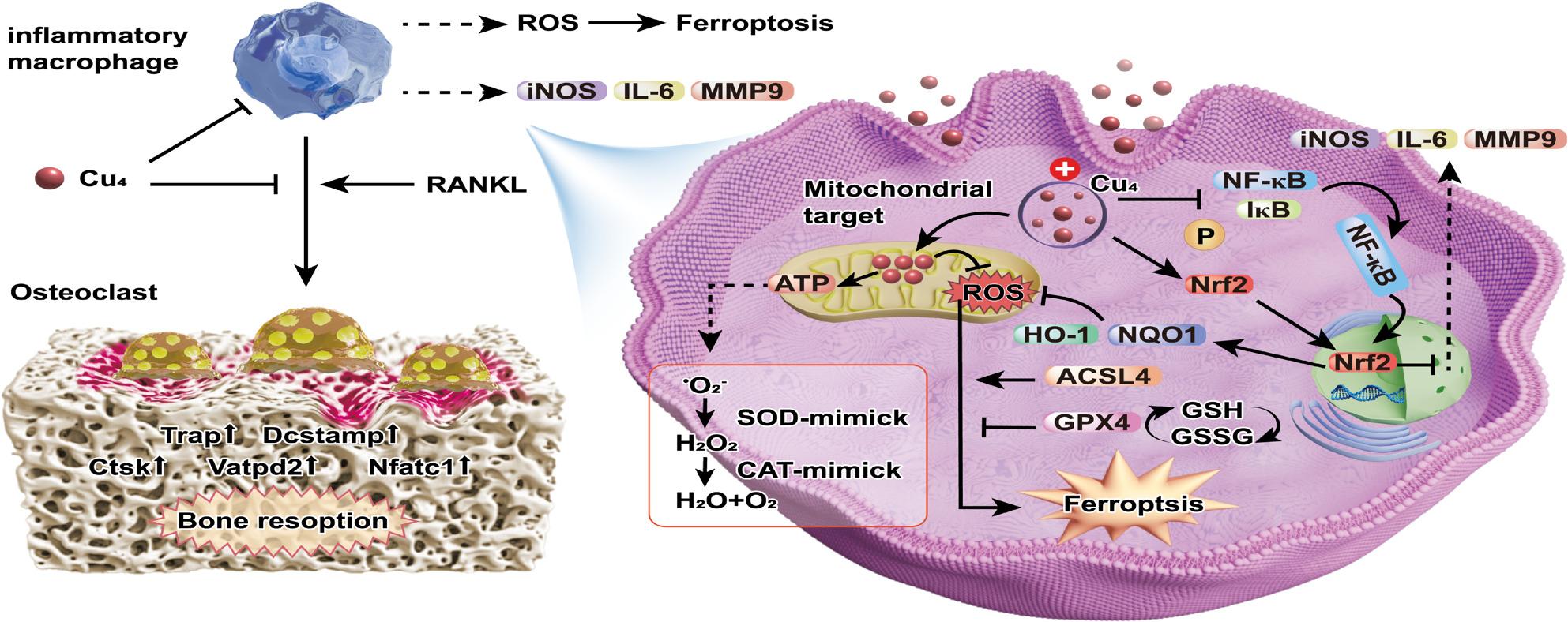
Novel ultrasmall nanoclusters (Cu₄) scavenge ROS, modulate Nrf2/NF-κB pathways, block ferroptosis and suppress osteoclastogenesis to alleviate inflammatory osteolysis.

**Supplementary Information:**

The online version contains supplementary material available at 10.1186/s12951-025-04009-2.

## Introduction

With the intensification of social aging, the incidence of arthritis has risen. Total joint replacement (TJR) is an exceptionally efficient surgical procedure targeting end-stage joint disorders [1]. Although it leads to enhancements in joint structure, functional ability, pain alleviation, and life quality, about 3–5% of patients continue to encounter postoperative complications like periprosthetic infection and aseptic loosening [2]. Inflammatory osteolysis is a grave complication arising after orthopedic procedures including TJR, mainly driven by persistent inflammatory reactions caused by prosthetic wear debris or bacterial substances such as lipopolysaccharides (LPS) [3]. It is marked by bone erosion at the bone-prosthesis interface and the infiltration of inflammatory cells, which seriously undermines the long-term stability of the prosthesis and may even require multiple revision surgeries [4]. At present, clinical treatment primarily depends on bisphosphonates to curb bone resorption and inflammation [5]. Nevertheless, prolonged use of these medications tends to result in side effects like gastrointestinal harm and osteoporosis, and they fail to concurrently halt the synergistic pathological processes involving inflammatory responses and osteoclast (OC) activation [6].

Macrophages, as the core regulatory cells in the pathological process of inflammatory osteolysis, are activated upon stimulation by wear particles or bacterial products, releasing substantial quantities of pro-inflammatory cytokines like TNF-α, IL-1β, and IL-6 [7]. Meanwhile, they can transform into OCs, the primary effector cells of osteolysis. These inflammatory cytokines can directly induce OC formation as well as synergize with RANKL to intensify OC maturation through the expression upregulation of RANKL and its receptor, forming a vicious cycle of “inflammatory amplification and enhanced bone resorption [8, 9].” Therefore, targeted regulation of macrophage function and related inflammatory pathways has become a key to blocking osteolysis.

In recent years, nanomedicines have drawn considerable interest owing to their capabilities in accurate targeting, minimized side effects, and flexible functional performance [10, 11]. Within the broad spectrum of nanomaterials, nanocluster is a highly potential class of ultrasmall nanoparticles, with sizes under 3 nm. In contrast to larger nanoparticles, nanoclusters possess consistent dimensions, precisely defined molecular structures, quantum size effects, strong stability and biocompatibility, and tunable structures, proving their huge application potential in biomedical fields. Importantly, nanoclusters containing variable valence metals at their cores demonstrate redox activities akin to nanozymes [12, 13], making them capable of well removing reactive oxygen species (ROS) and mimicking the roles of intracellular ROS-associated enzymes [14]. Over the past few years, a novel concept termed “clusterzymes” has been introduced into the scientific community. This innovative idea has garnered significant attention and interest, leading to an escalated exploration of nanoclusters. These nanoclusters are being meticulously investigated for their potential therapeutic applications, particularly in the realm of ROS-mediated neurodegenerative diseases. The increasing focus on nanoclusters as a potential treatment modality underscores the growing recognition of their unique properties and the promising avenues they open for advanced therapeutic interventions in these challenging medical conditions [15–19]. Despite the extensive exploration of gold and silver clusters, copper clusters have garnered comparatively less focus in tackling inflammatory and ROS-related diseases, even though copper is a crucial constituent of living organisms and a key metal component in natural enzymes [20–22].

**Fig. 1:**
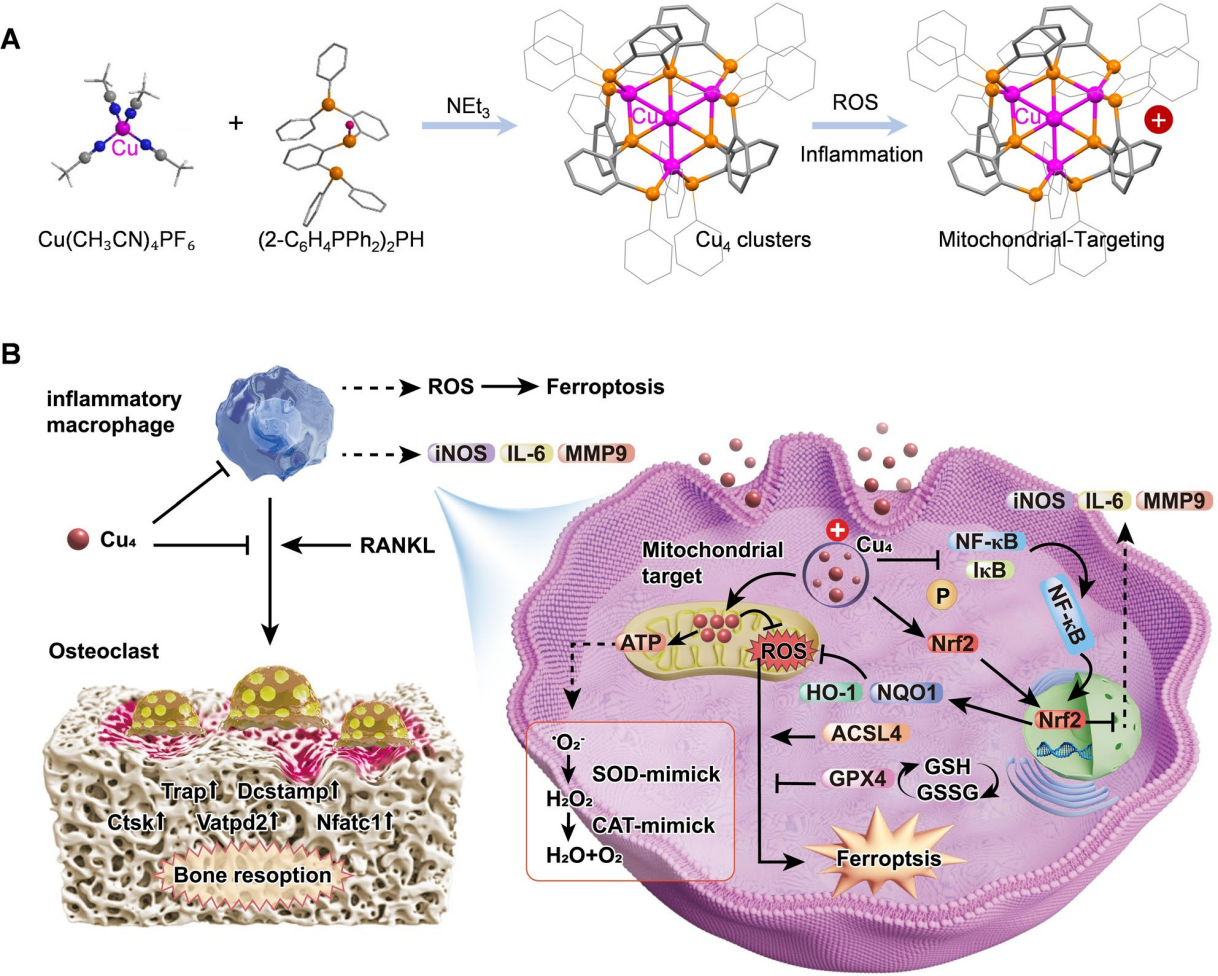
A schematic representation of the design and synthesis of Cu₄ clusters and their potential application for treating inflammatory osteolysis. (**A**) Cu₄ cluster synthesis. (**B**) Utilization of Cu₄ clusters for managing inflammatory osteolysis through leveraging their enzyme-mimetic activities, activation of the Nrf2 pathway, regulation of iron homeostasis and lipid peroxidation

In this study, we engineered Cu₄ clusters with the aim of achieving efficient therapeutic efficacy against inflammatory osteolysis, leveraging their distinctive structural and functional attributes (Fig. 1A). Cu₄ clusters exhibit enzyme-mimetic properties, as they are capable of mimicking superoxide dismutase (SOD) and catalase (CAT) for ROS scavenging [14]. Concomitantly, these clusters excel in activating the Nrf2 signaling pathway, accordingly inducing Nrf2 nuclear translocation and expression upregulation of endogenous anti-inflammatory and antioxidant molecules (e.g., HO-1 and NQO1) [23]. This regulatory effect subsequently inhibits the excessive activation of macrophages and the formation of OCs. Furthermore, Cu₄ clusters are able to modulate iron homeostasis and lipid peroxidation, thus blocking ferroptosis-associated pathological processes (Fig. 1B).

Many reported copper-based or noble metal clusterzymes exhibit either single enzyme-mimetic activity (e.g., only SOD-like or only CAT-like) or weak synergy between dual activities [24, 25]. In contrast, our Cu₄ clusters possess a well-defined core structure with uniform size - a feature that differs from amorphous Cu-based nanoparticles or larger noble metal clusters. This structural precision endows Cu₄ clusters with enhanced and synergistic SOD/CAT-like activities. And the mitochondrial targeting ability of Cu_4_ enables it to efficiently accumulate in mitochondria, the main source of ROS in cells, and exert its ROS scavenging function, making it more suitable for the treatment of inflammation and ROS-related diseases. While a few copper-based nanomaterials have been reported to modulate ferroptosis [26, 27], their mechanisms typically focus on a single pathway (e.g., only ROS scavenging or only GPX4 upregulation). Our Cu₄ clusters differ by targeting two core nodes of ferroptosis simultaneously: iron homeostasis regulation and Lipid peroxidation inhibition. This dual regulation is unique to our Cu₄ clusters, as it integrates their enzyme-mimetic ROS scavenging and Nrf2 pathway activation - a mechanism not reported in previous copper or noble metal clusterzymes.

Based on our research results, the Cu₄ cluster compounds exhibit a dual-pronged synergistic effect, providing a new molecular therapeutic strategy for treating inflammatory osteolysis diseases and other inflammation-related disorders.

## Experimental

### Reagents and antibodies

Escherichia coli-derived LPS was purchased in InvivoGen (San Diego, CA, USA). Minimal essential medium alpha (α-MEM) came from Gibco BRL (Gaithersburg, MD, USA). Fetal bovine serum (FBS) was provided by Avantor (Radnor, PA, USA). Recombinant mouse RANKL and macrophage colony-stimulating factor (M-CSF) came from R&D (Minneapolis, MN, USA). The Calcein/PI, cell counting kit (CCK)−8, cell cycle, dichloro-dihydro-fluorescein diacetate (DCFH-DA) and Hoechst 33,342 were provided by Beyotime Biotechnology (Shanghai, China). Dihydroethidium (DHE) was purchased from Med Chem Express (Shanghai, China). The C11-BODIPY probe was provided by Invitrogen (Carlsbad, CA, USA). FerroOrange Kit was purchased from Dojindo (Kumamoto, Japan). Primary antibodies against phospho-p65 (CST #3033), p65 (CST #8242), Nrf2 (CST #12721), HO-1 (CST #26416) and NQO1 (CST #62262) were provided by Cell Signaling Technology (Danvers, MA, USA). Primary antibodies against GPX4 (Abcam#ab125066), ACSL4 (Abcam#ab155282), iNOS (Abcam#ab283655) came from Abcam (Cambridge, UK). Primary antibodies against IL-6 (Affinity #DF6087), GAPDH (Affinity #AF7021) were provided by Affinity Biosciences (Cincinnati, OH, USA).

### Synthesis of Cu_4_ clusters

Under a nitrogen atmosphere, a mixture of (2-C_6_H_4_PPh_2_)_2_PH (100 mg, 0.18 mmol) and Cu(CH₃CN)₄PF₆ (90 mg, 0.24 mmol) in dichloromethane (DCM, 15 mL) received 5 min of stirring at room temperature (RT). After the addition of triethylamine (0.1 mL), an orange-red solution was formed, followed by another 2 h of stirring at RT to finish the reaction. The concentrated solution (about 2 mL) was added with diethyl ether to afford a solid precipitate. The solid was redissolved in DCM. Petroleum ether vapor was diffused into a DCM solution at RT over a three-day period to obtain the red crystals applying for X-ray diffraction (XRD). The synthetic yield of Cu₄ clusters is 60% and the purity is NMR grade.

### X-Ray crystallography

Single-crystal XRD data collection was achieved at 150(2) K on a Bruker D8 VENTURE diffractometer with a Mo Kα microfocus source (IµS 3.0, λ = 0.71073 Å) and a PHOTON II CPAD detector. Frames integrated using SAINT (v8.38 A) were absorption-corrected via multi-scan methods (SADABS) [28]. The structure was determined through intrinsic phasing (SHELXTL) and refined using full-matrix least-squares on F² (SHELXL-2018/3) via the ShelXle graphical interface (v4.8.6) [29].

### Electrochemical measurement

We conducted cyclic voltammetry (CV) using a PerkinElmer Model 263 A potentiostat/galvanostat in CH₂Cl₂ solutions with 0.1 M concentration (Bu^n^_4_N)(PF_6_) as supporting electrolyte. Measurements employed glassy carbon, platinum wire, and Ag/AgCl (3 M KCl) as working electrode, counter electrode and reference electrode, respectively (scan rate: 100 mV·s^− 1^).

### In vitro biocompatibility

First, the cytotoxicity of Cu_4_ was assessed using the CCK-8 assay. RAW 264.7 macrophages and bone marrow macrophages (BMMs) were seeded into 96-well plates (3 × 10^3^ cells/well) to undergo 12 h of culture to allow adherence. Subsequently, after the replacement of the culture medium with fresh medium containing (0–80 µg/mL) Cu_4_, the cells underwent 24–48 h of co-culture, followed by the replacement of the medium with fresh medium supplemented with CCK-8 to allow for 2 h of cell incubation at 37 ℃. A microplate reader was used for quantifying the cell viability at 450 nm wavelength.

Subsequently, the cytotoxicity of the Cu_4_ was re-evaluated using live/dead staining. We seeded RAW 264.7 macrophages in confocal dishes (1 × 10^4^ cells/dish). 12 h later, the cells received 24 h of treatment with 0–80 µg/mL Cu_4_. Finally, after calcein-AM and propidium iodide (PI) staining, the cells were imaged by virtue of confocal laser scanning microscopy (CLSM).

### Hemolysis assay

Red blood cells (RBCs) were harvested from the tail vein of mice and resuspended in saline. Cu_4_ at various concentrations were then mixed with RBC suspension, with saline serving as the negative control and double-distilled water as the positive control. All samples were incubated in a constant-temperature shaker at 37 °C and 100 rpm for 2 h, followed by 10 min centrifugation at 3500 rpm. After the transfer of 200 µL aliquot of the supernatant to a 96-well plate, the absorbance was measured at 545 nm for the calculation of the hemolysis rate.

### Cell cycle analysis

RAW 264.7 macrophages in 6-well plates (1 × 10^5^ cells/well) were added with culture medium containing Cu_4_ at various concentrations. 24 h culture later, the cells were harvested, fixed, stained with PI, and measured by flow cytometry (FCM).

### Cellular uptake assay

RAW 264.7 macrophages placed in confocal dishes underwent 6, 12, and 24 h co-culture with LPS (100 ng/mL) and Cu₄ clusters (20 µg/mL), respectively. Afterward, the cells received Hoechst 33,342 staining and were visualized using CLSM.

### Detection of intracellular ROS

With the objective of detecting intracellular ROS, we placed RAW 264.7 macrophages in confocal dishes to allow for 24 h of co-culture with 100 ng/mL LPS and 0, 5, or 20 µg/mL Cu₄ clusters. Afterwards, the cells were arranged to receive 30 min of incubation using DCFH-DA or DHE probes, followed by Hoechst 33,342 staining to label nucleus, and then were imaged via CLSM. Similarly, RAW 264.7 macrophages in 6-well plates were subjected to the same treatment as described above. Afterward, upon the collection of cells, the intracellular fluorescence intensities of DCF/DHE were detected via FCM.

### Western blotting

RAW 264.7 macrophages seeded in 6-well plates (1 × 10⁵ cells/well) underwent 24 h of treatment using 100 ng/mL LPS together with Cu_4_ clusters at various concentrations (0, 5, 10, and 20 µg/mL). Thereafter, cells were harvested for total protein extraction. The BCA Protein Assay Kit served for determining the total protein concentration in cell lysates. After 10% SDS-PAGE separation, proteins were moved onto 0.22 μm PVDF membranes. After being blocked with 5% BSA in 1× TBST, the membranes were incubated with primary antibodies (GAPDH, iNOS, IL-6, MMP9, P65, p-P65, ACSL4, GPX4, Nrf2, HO-1, NQO1, all 1:1000) for one night at 4 °C. These membranes then underwent incubation with horseradish peroxidase (HRP)-conjugated secondary antibodies, detected by virtue of Odyssey^®^ M Imaging System (LiCor Biosciences, USA).

### Real-time quantitative polymerase chain reaction (RT-qPCR) analysis

With the aim of quantifying the mRNA levels of pro-inflammatory and ferroptosis-related genes, we seeded RAW 264.7 murine macrophages into 6-well plates (5 × 10⁵ cells/well) to allow for 24 h of treatment in LPS (100 ng/mL) combined with Cu_4_ clusters (5, 10, or 20 µg/mL). BMMs in 6-well plates (2 × 10^5^ cells/well) received culture with M-CSF (30 ng/mL) and RANKL (100 ng/mL), concurrently treated with 2.5, 5, or 10 µg/mL Cu_4_ cluster concentrations over four days, aiming at assessing OC-related gene expression. The Axygen RNA Miniprep Kit was adopted for extracting total RNA as per the producer’s protocol. Reverse transcription was followed by RT-qPCR via TB Green™ Premix Ex Taq™ II. Table S1 lists primer sequences, with *Gapdh* set as the housekeeping gene.

### 1 Co-localization analysis of mitochondria and lysosomes

RAW 264.7 macrophages were placed in confocal dishes to receive 6, 12, and 24 h of co-culture and LPS (100 ng/mL) and Cu₄ clusters (20 µg/mL), respectively. Later, cells underwent half an hour of treatment with LysoTracker Green or Mito-Tracker Green at 37 °C. The last step was Hoechst 33,342 staining before visualization via CLSM.

### Mitochondrial ROS detection

The MitoSox Red probe was employed to evaluate mitochondrial ROS production. In short, RAW 264.7 macrophages in confocal dishes received 24 h of co-cultured with 100 ng/mL LPS and 0, 5, or 20 µg/mL Cu₄ clusters. Afterwards, the cells underwent 10 min of incubation using serum-free medium that contained 5 µM MitoSox Red probe at 37 °C. Finally, the cell nuclei underwent Hoechst 33,342 staining, and were imaged using CLSM.

### Mitochondrial membrane potential assay

Consistent with the protocol for mitochondrial ROS detection, following treatment with LPS and varying concentrations of Cu₄ clusters, the cells underwent 20 min of incubation using JC-1 working solution at 37 ℃. They were subsequently stained with Hoechst 33,342 and visualized using CLSM.

### Tartrate-resistant acid phosphatase (TRAP) staining assay

BMMs were plated in 96-well plates (5 × 10³ cells/well), followed by culture in medium supplemented with M-CSF (30 ng/mL) and RANKL (100 ng/mL), along with Cu_4_ clusters at 2.5, 5, and 10 µg/mL.The replacement of culture medium at an interval of 48 h persisted until OCs were observed on the fifth day. Following 15 min of 4% paraformaldehyde (PFA) fixation, the cells underwent 1 h of TRAP staining at 37 °C. OCs referred to TRAP-positive cells with ≥ 3 nuclei, with relevant pictures captured via a light microscope.

### bone resorption assay

BMMs were seeded on bone slices in 96-well plates at a density of 8 × 10^3^ cells/well, and cultured in complete ɑ‐MEM containing M‐CSF (30 ng/ml). 24 h later, the cells were stimulated with M‐CSF (30 ng/ml), RANKL (50 ng/ml), and different dose of Cu₄ clusters for 9 days. The OCs were then removed by mechanical agitation and sonication. The resorption pits were visualized under a scanning electron microscope. Pit areas were quantified using the ImageJ software.

### RNA sequencing (RNA-seq)

To investigate the mechanisms contributing to the anti-inflammatory activity of Cu_4_ clusters, RNA sequencing was conducted. In brief, RAW 264.7 macrophages in 6-well plates (1 × 10⁵ cells/well) were subjected to 24 h of incubation using 100 ng/mL LPS combined with 20 µg/mL Cu_4_ clusters. Upon the extraction of total RNA using TRIzol reagent, gene expression levels were quantified via Cosmos Wisdom (Hangzhou, China). For functional analysis, GO and KEGG pathway enrichment analyses were conducted utilizing Cosmos Wisdom Cloud (https://www.cwmda.com).

### Immunoprecipitation

For every sample, a total of 600 µg protein lysates were pre-incubated with IgA/G beads (Sigma, Shanghai, China). Anti-Keap1 antibody (Keap1, 1:50) and protein IgA/G beads were utilized to precipitate endogenous Keap1. Subsequently, the Keap1-Nrf2 immuno-complex was analyzed by Western blotting.

### Ferroptosis-related detection

The Ferro-orange probe was utilized to detect intracellular free Fe^2+^, while C11 BODIPY 581/591 probe served for assessing the intracellular lipid peroxidation level. RAW 264.7 macrophages in confocal dishes received 24 h of co-culture with 100 ng/mL LPS + 0, 5, or 20 µg/mL Cu₄ clusters. Afterward, the cells underwent half an hour of exposure to serum-free medium supplemented with either Ferro-orange or C11 BODIPY at 37 °C. Subsequently, after PBS rinse to eliminate excess probe, the cells were visualized via CLSM. Similarly, after undergoing the same treatment, RAW 264.7 macrophages were harvested, and stained using Ferro-orange or C11 BODIPY, with relevant images detected using FCM.

An MDA detection kit was adopted for measuring the intracellular malondialdehyde (MDA) content. RAW 264.7 macrophages in 6-well plates (1 × 10⁵ cells/well) received the co-treatment of 100 ng/mL LPS and Cu₄. The harvested cells were lysed via cytolysis buffer and sonication (3 s of ultrasonication, 10 s pause, repeated 30 times). The solution underwent centrifugation (12,000 g, 15 min) to yield the supernatant, with the absorbance of each group at 532 nm determined by virtue of a UV–vis.

### LPS-induced calvarial osteolysis model

The in vivo regulatory mechanism of Cu₄ clusters against inflammatory responses and bone resorption was assessed using the murine calvarial osteolysis model [30–32]. The Animal Care and Experiment Committee of the Ninth People’s Hospital Affiliated to Shanghai Jiao Tong University School of Medicine granted approval for the animal experiment (No. SH9H-2025-A1623-1). Briefly, twenty 6-week-old male C57/BL6 mice fell into four groups in a random manner: (1) Sham group (injected with saline); (2) LPS group (injected with a saline solution containing 10 mg/kg body weight LPS); (3) Low-dose Cu₄ group (Injected with 10 mg/kg body weight LPS + 5 µg/mL Cu₄ clusters simultaneously); (4) The high-dose Cu₄ group (Injected with 10 mg/kg body weight LPS + 20 µg/mL Cu₄ clusters simultaneously). After anesthetizing mice, we implanted 4 mm × 4 mm × 2 mm collagen sponges in PBS or LPS at the midline of the skull. Three days later, all mice received subcutaneous injections at the calvarial site every other day for an additional 11 days. The termination of the experiment was followed by the euthanasia of the mice. The entire calvaria was harvested to receive saline rinse and 48 h of fixation in 4% PFA in succession for subsequent analyses.

### In vivo biodistribution assessment

To evaluate the biodistribution and metabolic profile of Cu₄ clusters in major organs, 6-week-old male C57/BL6 mice (*n* = 3) received subcutaneous injections of calvarias of Cu₄ clusters at a concentration of 200 µg/mL in a volume of 50 µL. The animals were euthanized 48 h post-injection, and key organs were collected for analysis. Tissue samples were weighed, homogenized, and digested in aqua regia. Copper content in each sample was quantified by inductively coupled plasma mass spectrometry (ICP-MS), and the results were expressed as the percentage of injected dose per gram of tissue (%ID/g).

### Statistical analysis

Data analysis relied on GraphPad Prism 9.0 software. All data were expressed as mean ± standard deviation (SD). For between-group comparison, a two-tailed unpaired Student’s t-test was applied following a homogeneity of variance test. One-way ANOVA with Tukey’s post hoc test served for multiple group comparison. Statistical significance was defined as *p < 0.05, **p < 0.01, and ***p < 0.001.

## Results and discussion

### Cu_4_ cluster synthesis and characterization

**Fig. 2:**
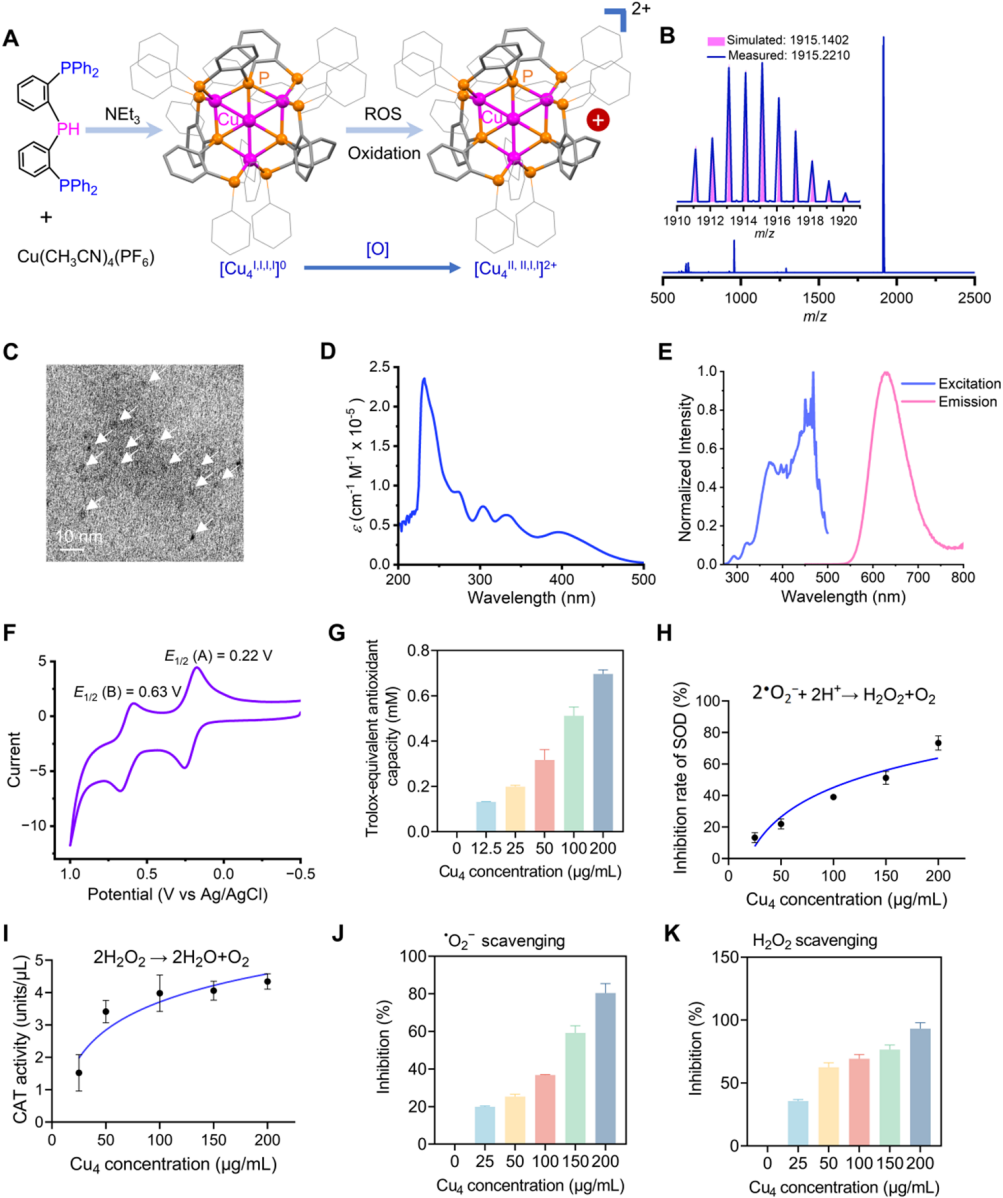
Cu_4_ cluster synthesis and characterization. (**A**) Synthetic route of Cu_4_ cluster. (**B**) The plot of high-resolution mass spectrometry (HR-MS) of Cu_4_ cluster. (**C**) TEM image of the Cu_4_ cluster. (**D**) The UV-Vis absorption spectrum of Cu_4_ cluster in CH_2_Cl_2_ solution at the concentration of 2 × 10 − 5 M. (**E**) The photoluminescent (PL) spectrum of Cu_4_ cluster in CH_2_Cl_2_ solution at the concentration of 2 × 10 − 5 M upon excitation at 398 nm. (**F**) The cyclic voltammogram of Cu_4_ cluster in CH_2_Cl_2_ (0.1 M (Bun4N)(PF6)) solution. (**G**) ABTS^·+^ scavenging capacity. (**H**) SOD-mimetic activity. (**I**) CAT-mimetic activity. (**J**) Superoxide anion (^•^O_2_^−^) scavenging. (K) Hydrogen peroxide (H_2_O_2_) scavenging capacity

Cu_4_ crystals were obtained by diffusion into a dichloromethane solution and the crystal structure was determined by X-ray diffraction (Fig. 2A). Single-crystal XRD analysis demonstrated the crystallization of Cu₄ in the *P-*3c1 space group of the trigonal crystal system. Cationic Cu_4_ cluster ([Cu_4_L_3_](PF_6_)) is composed of four cuprous ions and three bis(2-(diphenylphosphaneyl)phenyl)phosphane (HL) ligands, where three cuprous ions forms a equilateral triangle whereas another cuprous embedded at the center of the triangle plane. The Cu₄ cluster was characterized by ESI-MS (Fig. 2B), ^1^H NMR (Fig. S1) and ^31^P NMR spectra (Fig. S2). The ESI-MS showed a distinct set of isotopic peaks, corresponding to [Cu_4_L_3_]⁺ (simulated (Sim.) m/z = 1915.1402, experimental (Exp.) m/z = 1915.2210). The purity of Cu_4_ was also clearly evidenced by the powder X-ray diffraction (PXRD), showing the measured XRD coincide closely with those obtained through single-crystal XRD analysis (Fig. S3).

Transmission electron microscopy (TEM) revealed that Cu_4_ clusters possess a uniform nanomorphology (diameter: 2.50 ± 0.79 nm) (Fig. 2C, S4). We measured the Zeta potential of Cu_4_ before and after ROS treatment, and the results showed that under 5% H_2_O_2_ stimulation, the Zeta potential of Cu_4_ significantly shifted to a positive value of 3.67 mV. This indicates that Cu_4_ can acquire a positive charge in the inflammatory microenvironment, which then enables electrostatic attraction to the negatively charged mitochondrial matrix (Fig. S5). The UV–Vis absorption spectrum of Cu_4_ clusters in CH_2_Cl_2_ exhibits 3 distinct bands at 305, 332, and 396 nm (Fig. 2D), demonstrating an obvious green luminescence band at about 613 nm with a microsecond lifetime (19.43 µs) in CH_2_Cl_2_ when excited at > 240 nm (Fig. 2E), which allows for directly monitoring the cellular transportation pertaining to Cu_4_ clusters.

To evaluate the stability of Cu₄ cluster in biological-relevant environments, we incubated Cu₄ cluster in 10% fetal bovine serum (FBS, mimicking in vivo protein-rich conditions) and phosphate-buffered saline (PBS, mimicking physiological ionic environment). The results (Fig. S6) showed that at all time points (0–7 days), Cu₄ clusters maintained a uniform spherical morphology without obvious aggregation, fragmentation, or precipitation, and the average diameter of Cu₄ cluster remained stable. The stability of Cu₄ in serum was further examined by UV-Vis analysis (Fig. S7). The absorption profiles of the Cu₄ clusters exhibited negligible spectral variations over time, which also indicates their high stability in biological media.

Cu_4_ redox properties were examined by CV in acetonitrile solutions. As shown in the Fig. 2F, two pairs of reversible redox waves are visible, corresponding to *E*_1/2_ (A) = 0.22 V (for the [Cu_4_^I, I,I, I^]^+^/[Cu_4_^II, I,I, I^]^2+^ redox process) and *E*_1/2_ (B) = 0.63 V (for the [Cu_4_^II, I,I, I^]^2+^/[Cu_4_^II, II, I,I^]^3^⁺ redox process). Since the Cu_4_ cluster becomes a fully charge-delocalized system upon oxidation, the oxidation states of the four Cu atoms in mixed-valance state should be averaged. Consequently, in the first oxidation product [Cu_4_^II, I,I, I^]^2+^, the average oxidation state per Cu atom is 5/4 (+ 1.25), while in the second oxidation product [Cu_4_^II, II, I,I^]^3^⁺, the average oxidation state per Cu atom is 6/4 (+ 1.5). The XPS (Fig. S8) shows that for Cu_4_^I, I,I, I^, the Cu(2p_1/2_) and Cu(2p_3/2_) peaks appear at 951.9 eV and 931.8 eV, respectively, which are close to the binding energy of Cu(I). In striking contrast, the Cu(2p_1/2_) and Cu(2p_3/2_) peaks were respectively observed at 953.4 eV and 933.4 eV for one-election oxidized Cu_4_^II, I,I, I^ cluster, indicating a significant increase in the positions of the Cu(2p_1/2_) and Cu(2p_3/2_) peaks relative to those of Cu_4_^I, I,I, I^. However, the binding energy of Cu(2p_3/2_) peak in one-election oxidized Cu_4_^II, I,I, I^ cluster still deviates obviously from that for typical Cu(II) (934.3 eV). Thus, the valence in one-electron oxidized product [Cu_4_^II, I,I, I^](PF_6_)_2_ cluster is better described as an average valence of + 1.2 for each Cu atom, where the one-electron oxidation is averagely distributed on Cu_4_ cluster, not localized at anyone of the four Cu atoms. In fact, [Cu_4_^II, I,I, I^]^2+^ belongs to a totally electronic delocalization mixed-valence state. Taken together, the CV and XPS of the Cu_4_ cluster reveals its capacity for two-step electron transfer and reversible redox processes.

### Cu_4_ clusters exhibit enzyme-like and ROS scavenging activities

Building on the promising electrochemical properties of Cu_4_ clusters, we investigated their potential enzymatic activities. Total antioxidant capacity, assessed via the ABTS assay, revealed dose-dependent enhancement of antioxidant activity (Fig. 2G). Evaluation of SOD-like activity using the WST-8 method demonstrated significant superoxide dismutase-mimetic behavior, evidenced by concentration-dependent suppression of formazan formation (Fig. 2H). Given the role of SOD in catalyzing superoxide anion (^•^O_2_^−^) disproportionation to hydrogen peroxide (H_2_O_2_) and oxygen (O_2_), we subsequently examined CAT-like activity. Cu_4_ clusters exhibited CAT-mimetic activity, catalyzing the oxygenation of colorimetric substrates in peroxidase-coupled reactions (Fig. 2I). Further analysis of ROS scavenging capacity showed potent, dose-dependent elimination of ^•^O_2_^−^ and H_2_O_2_ (Figs. 2J, K). At 200 µg/mL, Cu_4_ clusters scavenged 80.43% of ^•^O_2_^−^ and 93.17% of H_2_O_2_. Collectively, these results establish Cu_4_ clusters as potent multienzyme mimics with synergistic SOD- and CAT-like activities, enabling efficient ROS scavenging [7, 33, 34].

### Biocompatibility of Cu₄ clusters

**Fig. 3:**
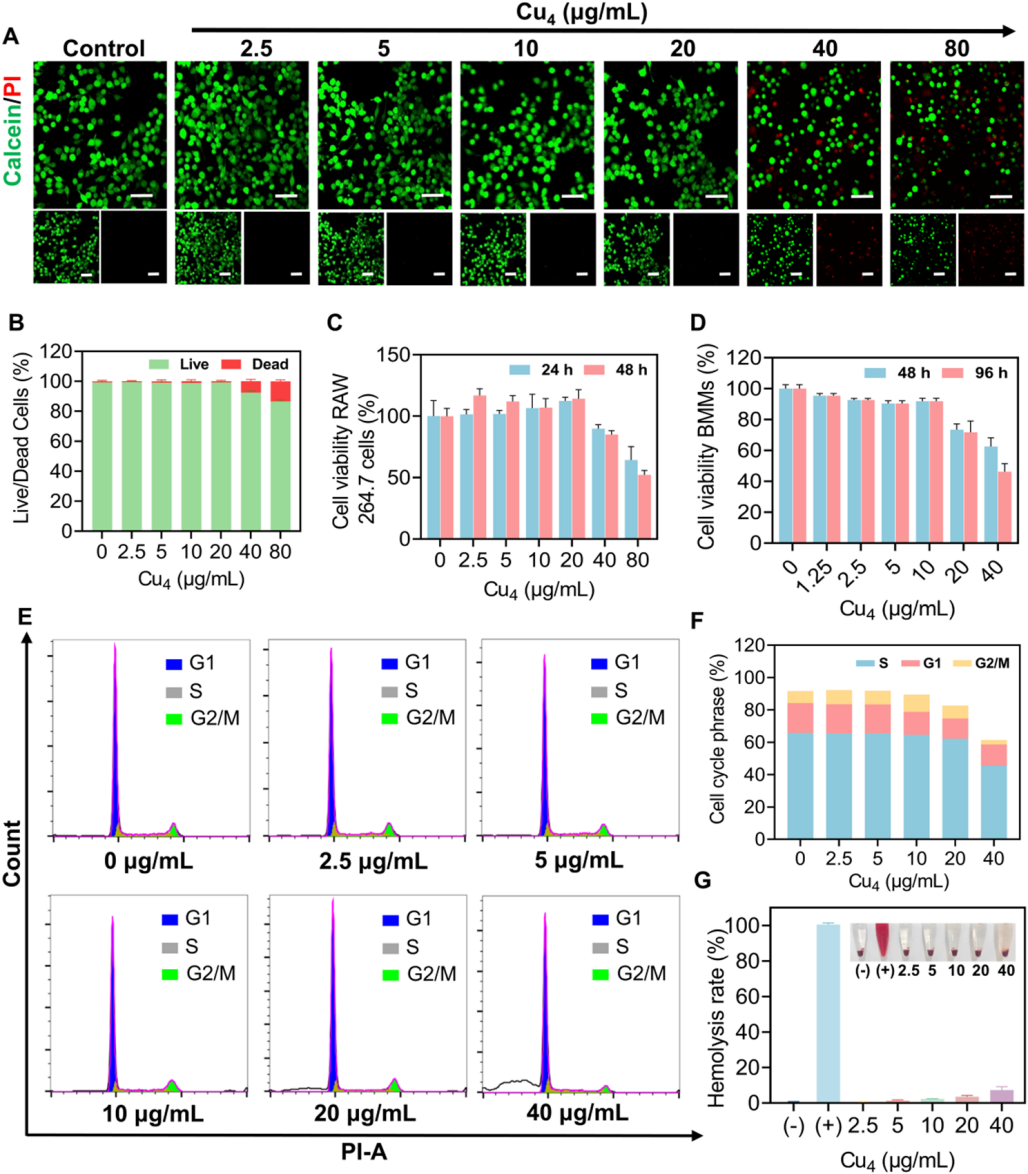
Biocompatibility of Cu₄ clusters. (**A**) Live/dead staining of RAW 264.7 macrophages after 24 h of Cu₄ cluster treatment at varying concentrations. (**B**) Quantitative analysis of calcein-AM positive cells and PI positive cells. (**C**) Viability of RAW 264.7 macrophages following 24 and 48 h of culture with varying concentrations of Cu₄ clusters. (**D**) Viability of mouse BMMs following 48 and 96 h of culture with varying concentrations of Cu₄ clusters. (**E**) Cell cycle analysis of RAW 264.7 macrophages following 24 h of Cu₄ cluster incubation at various concentrations. (**F**) Quantitative analysis of cell cycle. (**G**) Hemolysis assay of Cu₄ clusters. Data represent the mean ± SD, *n* = 3, Scale bar = 50 μm

Biocompatibility represents a fundamental prerequisite for the development of multifunctional nanoagents like Cu₄ clusters. To systematically evaluate the safety profile of Cu₄ clusters, a series of experiments was conducted in evaluating the biocompatibility of Cu₄ clusters. According to live/dead staining, at 0–20 µg/mL, red fluorescence was negligible (Fig. 3A). However, the emergence of PI-positive cells was observed at 40 µg/mL (approximately 7.5%), and the proportion of dead cells further escalated to 13.5% at 80 µg/mL (Fig. 3B). Next, CCK-8 assays served for determining Cu₄ clusters-dependent viability alternation of RAW 264.7 macrophages at 24 and 48 h. At 40 µg/mL, the cell viability was 89.87% and 85.02% relative to the control group, respectively. Upon treatment with 80 µg/mL Cu₄ clusters for 48 h, the cell viability decreased to only 32.23% (Fig. 3C). These observations suggest that the safe concentration range of Cu₄ clusters for RAW 264.7 macrophages was 0 ~ 20 µg/mL. Similarly, we evaluated the safety of Cu₄ clusters on BMMs, and the results demonstrated that the cell viability remained above 90% at 10 µg/mL or below, whether treated for 48–96 h (Fig. 3D). Consistently, flow cytometric analysis showed that Cu₄ clusters exerted only a negligible influence on cell cycle distribution at 20 µg/mL (Fig. 3E, F). To further investigate whether Cu₄ clusters disrupt blood cell homeostasis, we conducted hemolysis assays, where RBCs underwent incubation using different concentrations of Cu₄ clusters. According to Fig. 3G, 20 µg/mL Cu₄ clusters caused a slight reduction in RBC viability, with the hemolysis rate remaining below 4%.

### Cellular uptake and anti-inflammatory effects of Cu₄ clusters

**Fig. 4:**
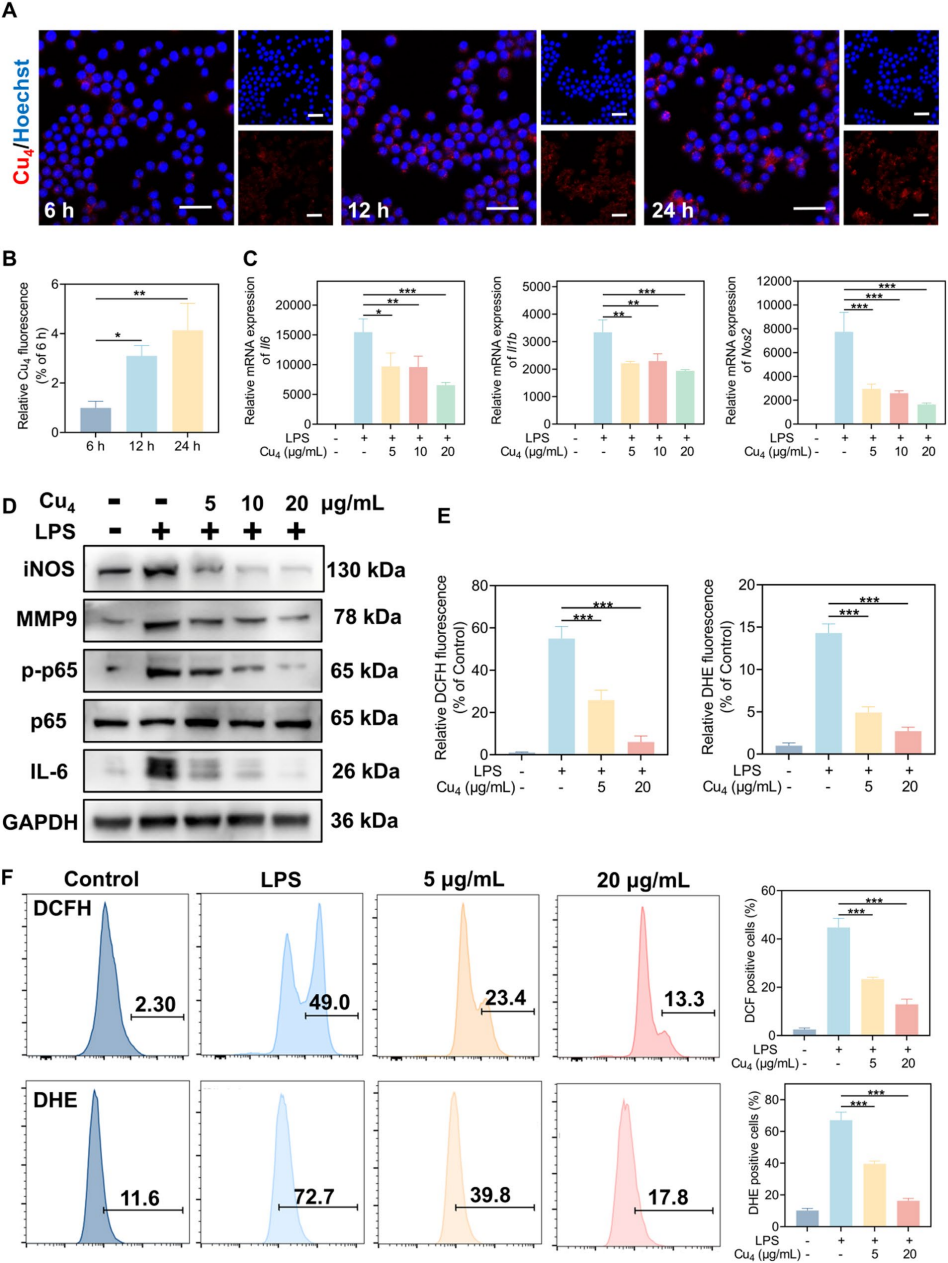
Cellular uptake and anti-inflammatory effects of Cu₄ clusters. (**A**) The cellular uptake of Cu₄ clusters at 6 h, 12 h, and 24 h. (**B**) Quantitative analysis of Cellular uptake of Cu₄ clusters. (**C**) RT-qPCR analysis of pro-inflammatory gene expression in RAW 264.7 macrophages after Cu₄ cluster treatment at varying concentrations. (**D**) WB analysis of iNOS, MMP9, p-p65, p65, and IL-6 in RAW 264.7 macrophages following Cu₄ cluster treatment at varying concentrations. Quantitative analysis of (**E**) fluorescence intensity of DCFH-DA and DHE, and (**F**) the percentage of DCFH-DA and DHE positive cells using FCM. Data are in the format of the mean ± SD, *n* = 3, Scale bar = 50 μm, *p < 0.05; **p < 0.01; ***p < 0.001

CLSM assisted in examining the cellular uptake of Cu₄ clusters at 6, 12, and 24 h. Blue fluorescence denoted cell nuclei, while red fluorescence signified the presence of Cu₄ clusters. Accordingly, the intensity of red fluorescence within RAW 264.7 cells slowly increased over time. By 24 h, prominent red fluorescence was observable in the cells, confirming that Cu₄ clusters could be efficiently internalized by RAW 264.7 cells, thus laying a foundation for subsequent investigations into the intracellular behaviors of Cu₄ clusters (Fig. 4A, B).

Motivated by the favorable biocompatibility and cellular internalization characteristics of Cu₄ nanoclusters, we subsequently probed their potential anti-inflammatory efficacy. Specifically, RAW 264.7 macrophages were exposed to LPS alongside 5, 10, and 20 µg/mL Cu₄ clusters for a 24-h duration. For the assessment of how Cu₄ clusters affected LPS-induced proinflammatory gene transcription, we employed RT-qPCR for *Il6*,* Il1b*,* and Nos2* mRNA level measurement. According to Fig. 4C, LPS stimulation (LPS+, Cu₄-) robustly upregulated the mRNA expression of *Il6*,* Il1b*,* and Nos2*. However, co-treatment with Cu₄ clusters dose-dependently suppressed these LPS-induced transcriptions. These cytokines and enzymes are key mediators of the inflammatory response, and their transcriptional dynamics reflect the activation status inflammatory-related signaling pathways [35, 36]. Cu₄ clusters also exerted a dose-dependent inhibitory effect on these LPS-induced protein expressions. As shown in Fig. 4D and S9, at 5 µg/mL Cu₄, a slight reduction in iNOS, MMP9, and IL-6 protein levels was observed. At 10 and 20 µg/mL, the protein abundances of these inflammatory markers were further decreased. For instance, iNOS protein, which was highly induced by LPS, was almost undetectable at 20 µg/mL Cu₄. The phosphorylation level of p65, crucial for NF-κB-mediated inflammatory gene transcription, was also dose-dependently suppressed by Cu₄ clusters, while the total p65 protein level remained relatively unchanged across groups. These findings conform to the mRNA expression data, collectively demonstrating that Cu₄ clusters inhibit LPS-triggered inflammatory responses in RAW 264.7 macrophages through restricting both the NF-κB signaling pathway and the process that pro-inflammatory proteins are produced.

Macrophages, upon activation by diverse inflammatory stimuli, experience respiratory bursts meanwhile generating excessive ROS, triggering oxidative stress. This oxidative stress might more deeply worsen inflammatory responses, maintaining a vicious cycle [37, 38]. Hence, we examined how Cu₄ clusters affected the ROS overproduction in inflammatory macrophages, utilizing DCFH and DHE probes. Fig. S10 and S11 demonstrate prominent green/red fluorescence in the LPS-treated group. Cu₄ clusters treatment significantly diminished the fluorescence intensity, confirming that Cu₄ clusters can effectively mitigate LPS-induced ROS overproduction. According to Fig. 4E, Cu_4_ clusters at varying doses greatly reduced the number of DCFH-positive and DHE-positive cells. Particularly at 20 µg/mL, Cu₄ clusters decreased DCFH-positive or DHE-positive cell counts to 10.9% or 18.9% of those observed under LPS stimulation alone, respectively. The results of FCM also verified this conclusion quantitatively (Fig. 4F). Collectively, Cu_4_ clusters well restrict inflammation-induced oxidative stress and restores intracellular ROS homeostasis through its potent ROS scavenging and enzyme-mimicking activities.

We also compared Cu₄ clusters and Cu(OAc)_2_ in LPS-activated macrophages using immunofluorescence and qPCR (Fig. S12, S13). The results indicate that unlike Cu₄ clusters, Cu(OAc)_2_ is unable to effectively alleviate the excessive production of ROS induced by LPS or inhibit the expression of inflammatory factors. This suggests that the nanocluster structure is essential for the anti-inflammatory and antioxidant effects of Cu₄ clusters.

### Cellular distribution and protective mechanism of Cu_4_ clusters against mitochondria

**Fig. 5:**
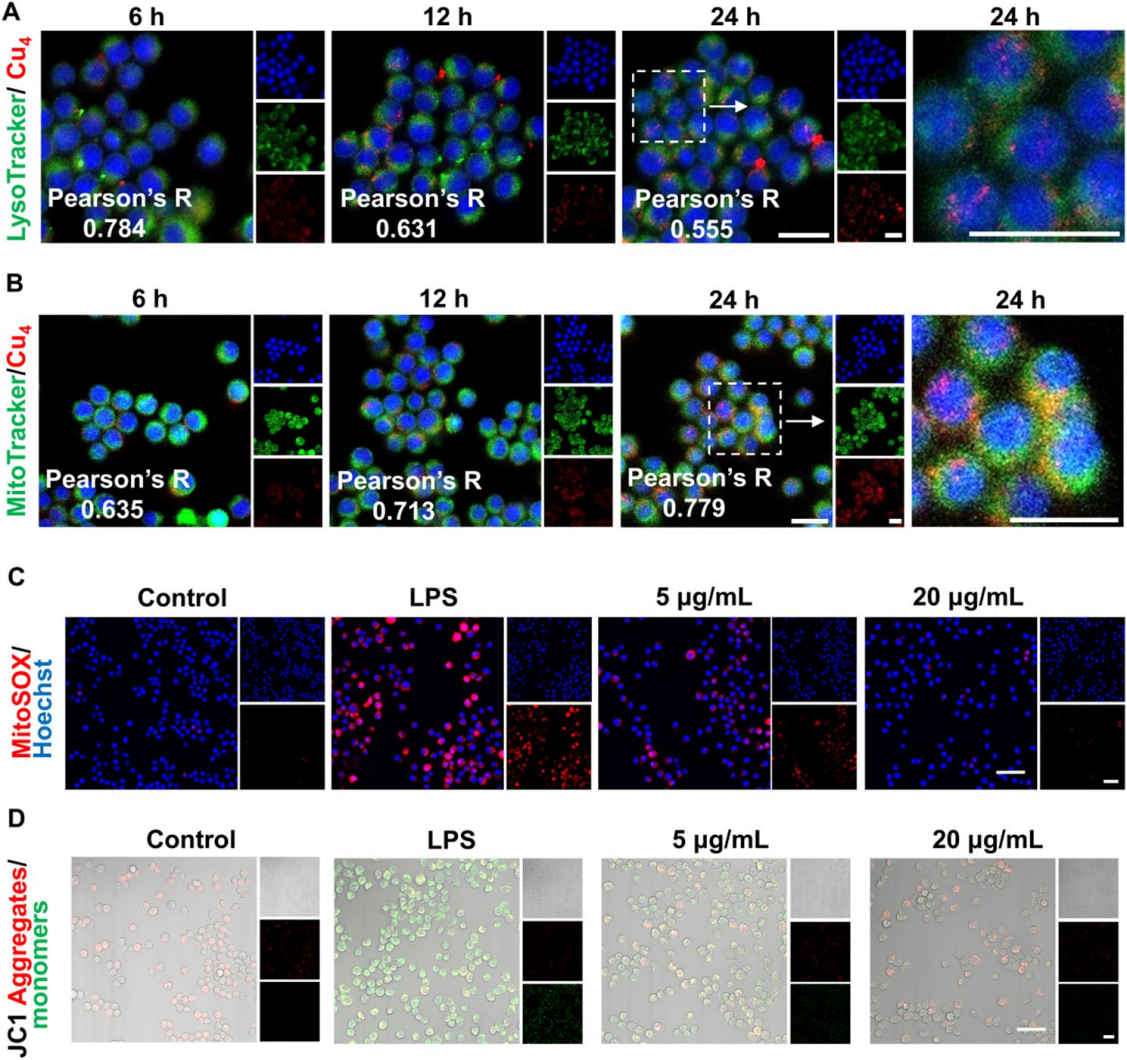
Cellular distribution and protective mechanism of Cu₄ clusters against mitochondria. The typical confocal image illustrating the co-localization of Cu₄ clusters with (**A**) lysosomes, and (**B**) mitochondria. Confocal images and quantitative analysis of RAW 264.7 macrophages after (**C**) Cu₄ cluster treatment and MitoSOX staining, and (**D**) Cu₄ clusters treatment and JC-1 mitochondrial membrane potential probe staining. Data are in the format of the mean ± SD, *n* = 3, Scale bar = 50 μm, *p < 0.05; **p < 0.01; ***p < 0.001

Fluorescence imaging was utilized to probe the time-dependent cellular distribution of Cu₄ clusters. Red fluorescence denotes Cu₄ clusters, green fluorescence represents either lysosomes or mitochondria, and blue fluorescence indicates cell nuclei. When Cu₄ clusters colocalize with an organelle, yellow fluorescence emerges as a result of the overlap of green and red fluorescence. According to Fig. 5A and B, the red fluorescence intensity within cells gradually increases over time. Intriguingly, from 6 h to 24 h, the colocalization of Cu₄ clusters with lysosomes gradually diminishes, while their colocalization with mitochondria progressively strengthens. This time-varying intracellular distribution pattern indicates that Cu₄ clusters undergo active trafficking within cells and tend to localize to mitochondria as time elapses. The changes in the Pearson’s correlation coefficient corroborate this finding; the closer the coefficient is to 1, the stronger the colocalization of red and green fluorescence. In inflammatory osteolysis, mitochondrial dysfunction is a pivotal upstream event that amplifies oxidative stress, activates NF-κB signaling, and promotes osteoclast differentiation. Thus, verifying whether Cu₄ clusters’ mitochondrial localization translates to functional protection of mitochondria is critical to linking their spatial distribution to therapeutic efficacy.

Mitochondria are one of the main sites for intracellular ROS production. During the inflammatory response, mitochondrial dysfunction can lead to a massive generation of ROS [39, 40]. The mitochondrial enrichment function of Cu₄ is beneficial for it to directly act on the core of oxidative stress, thereby enhancing its anti-inflammatory efficacy. To further decipher the functional implications of Cu₄ clusters’ mitochondrial localization in anti-inflammatory responses, we assessed mitochondrial ROS (mtROS) and membrane potential (MMP) using MitoSOX and JC-1 staining, respectively. As shown in Fig. 5C and S14, there was little red fluorescence in mitochondria of the control group. LPS treatment dramatically enhanced the red fluorescence. After Cu₄ treatment, the ROS level in mitochondria decreased significantly. This attenuation of mtROS aligns with Cu₄ clusters’ mitochondrial targeting (evidenced by colocalization trends), suggesting that their accumulation in mitochondria directly intercepts ROS overproduction - a key driver of inflammatory amplification. JC-1 staining (red = healthy MMP, green = depolarized MMP) revealed that LPS disrupted mitochondrial integrity, shifting fluorescence from red to green (monomeric JC-1). Conversely, Cu₄ clusters restored MMP: at 5 µg/mL, partial red fluorescence recovery indicated initial membrane potential stabilization; at 20 µg/mL, prominent red fluorescence signaled robust MMP preservation (Fig. 5D, S15). This protective effect on mitochondrial function is synergistic with Cu₄’s mitochondrial localization - by maintaining membrane potential [41, 42], Cu₄ clusters safeguard ATP production and mitochondrial signaling, which are critical for resolving inflammation. In conclusion, Cu₄ clusters exhibit a time-dependent cellular distribution pattern with a tendency to localize to mitochondria as well as protect against mitochondria through modulating mtROS levels and maintaining MMP, thereby contributing to their anti-inflammatory effects at the cellular level.

### Impact of Cu₄ clusters on OC formation, fusion, and function

**Fig. 6:**
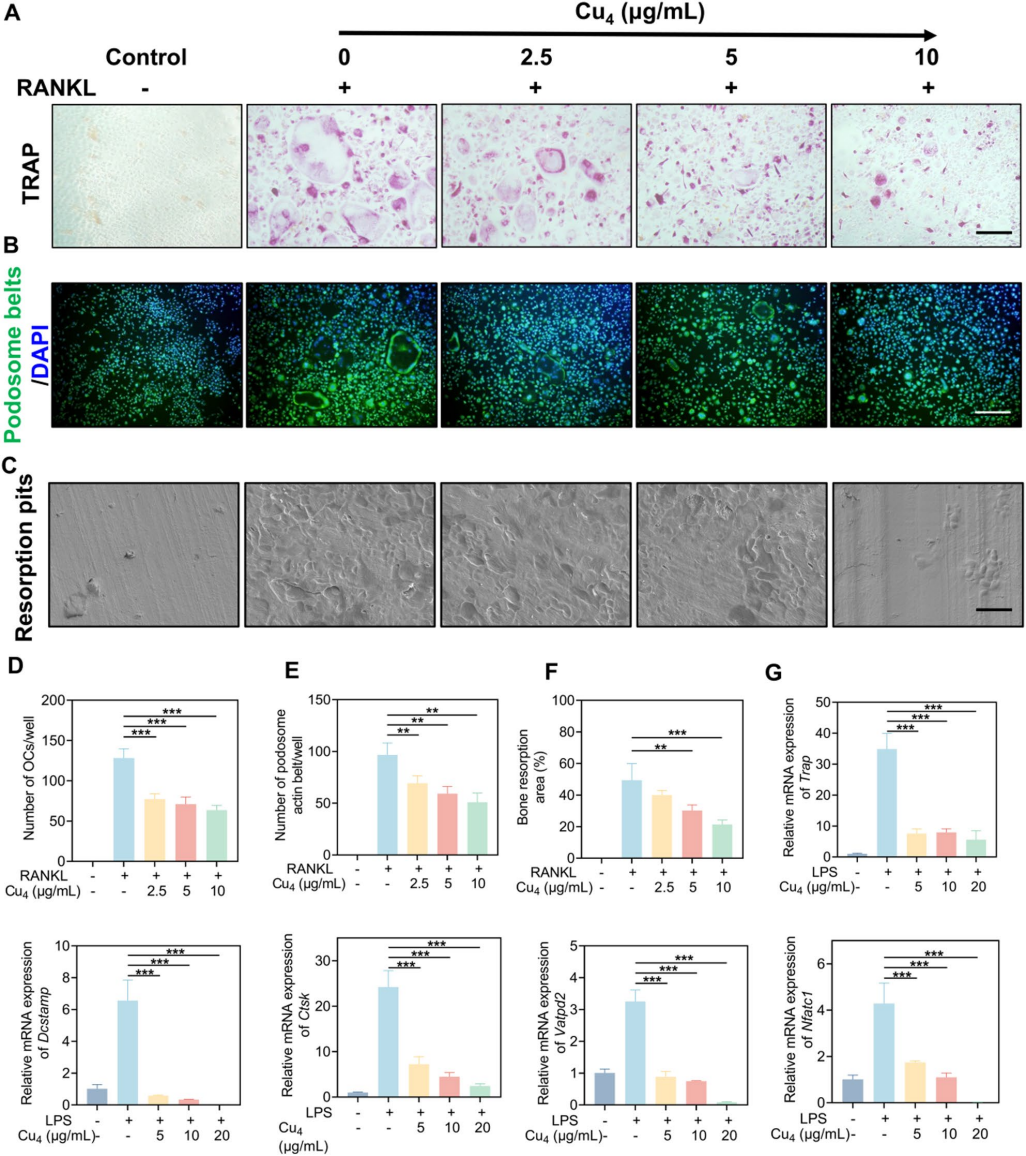
Impact of Cu₄ clusters on OC formation, fusion, and function. (**A**) Representative TRAP staining images of BMMs. (**B**) Representative immunofluorescence images of podosome actin belts in BMMs. (**C**) Representative SEM images of bone resorption pit assays. (**D**) Number of OCs per well. (**E**) The number of podosome actin belts per well. (**F**) Bone resorption area (%). (**G**) RT-qPCR analysis of OCs-related gene expression in BMMs. Data are in the format of the mean ± SD, *n* = 3, Scale bar = 100 μm, *p < 0.05; **p < 0.01; ***p < 0.001

OCs are important somatic cells capable of mediating bone resorption, and their abnormal activation (driven by inflammatory signals and RANKL) is the terminal effector event of inflammatory osteolysis. Thus, after confirming Cu₄ clusters’ anti-inflammatory efficacy, investigating their direct regulatory effects on OC biology is critical to verifying whether the clusters can target both “inflammatory initiation” and “bone resorption execution” - a dual intervention that addresses the core pathological cascade of osteolysis. We selected a 10 µg/mL maximum concentration for Cu_4_ clusters, as determined by testing their cytotoxicity in BMMs. TRAP staining (Fig. 6A, D) revealed that, RANKL induced the differentiation of BMMs into large, multinucleated TRAP-positive OCs with intense purple staining; yet, Cu₄ clusters dose-dependently inhibited this process. TRAP is a well-validated phenotypic marker of mature OCs, and multinucleation is a hallmark of OC functional competence. The dose-dependent reduction in TRAP-positive multinucleated cells directly confirms that Cu₄ clusters suppress OC differentiation - a key step in blocking bone resorption, as immature mononuclear OCs lack bone-resorbing activity. Immunofluorescence for podosome actin belts also showed that robust, continuous podosome belts formed in RANKL-stimulated controls, while Cu₄ clusters disrupted belt formation (Fig. 6B and E). The scanning electron microscope (SEM) images showed that after RANKL treatment, the bone resorption pits became significantly more prominent, while Cu₄ clusters was able to reverse this change (Fig. 6C and F). For further elucidating the function of Cu₄ clusters in OCs, we conducted RT-qPCR to assess the expression of OC-associated genes: *Trap* (OC differentiation), *Dcstamp* (OC fusion), *Ctsk* (bone resorption), *Vatpd2* (bone resorption), and *Nfatc1* (regulating osteoclastogenesis). As anticipated, Cu₄ clusters potently downregulated the gene expression (Fig. 6G). Collectively, these results complement the clusters’ anti-inflammatory effects: Cu₄ clusters not only reduce inflammatory signals that drive OC activation ("Cellular uptake and anti-inflammatory effects of Cu₄ clusters") but also directly suppress OC differentiation, fusion, and resorptive function.

### Transcriptomic analysis of Cu₄ clusters in inflammatory macrophages

**Fig. 7:**
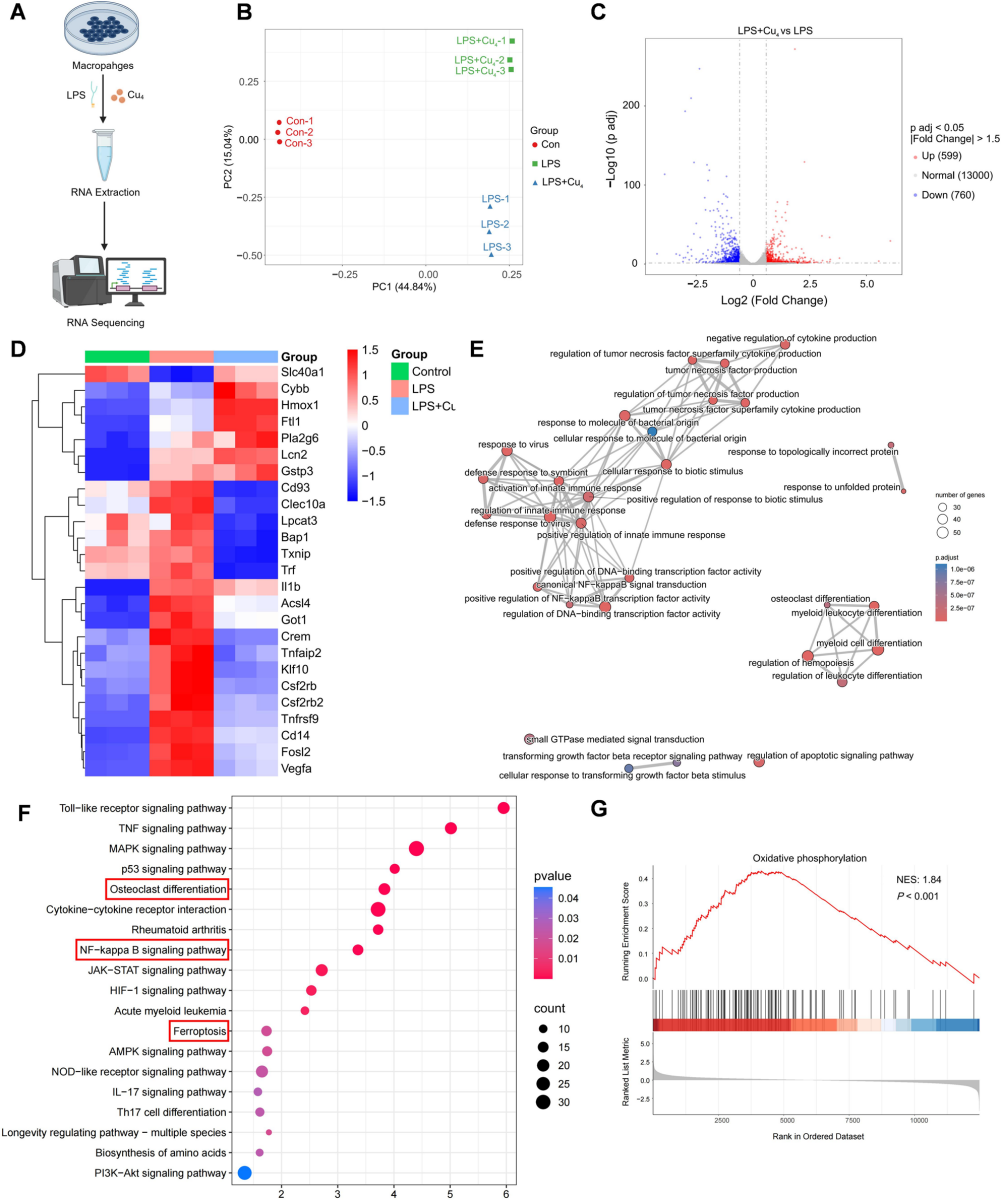
Transcriptomic analysis of Cu₄ clusters in inflammatory macrophages. (**A**) Schematic diagram of RNA sequencing. (**B**) PCA of the transcriptomic profile. (**C**) Volcano plots illustrating the DEGs between the LPS and LPS + Cu_4_ groups. (**D**) Clustered heatmap demonstrating the representative inflammation and oxidative related genes (fold change ≥ 1.5 and *P* < 0.05). (**E**) Emapplot of GO enrichment analysis, (**F**) KEGG pathway enrichment analysis, and (G) GSEA of the DEGs between the LPS and LPS + Cu₄ groups

To elucidate the anti-inflammatory mechanism of Cu₄ clusters, we conducted RNA sequencing on Cu_4_ cluster-treated inflammatory RAW 264.7 macrophages (Fig. 7A). According to principal component analysis (PCA), different transcriptomic profiles were observed among the control, LPS-stimulated, and LPS + Cu_4_ groups (Fig. 7B). In volcano plot analysis, 3529 genes underwent obvious upregulation and 3617 genes underwent obvious downregulation in the LPS group versus the control (Fig. S16). Notably, Cu_4_ cluster treatment resulted in 599 genes with obvious upregulation and 760 genes with obvious downregulation relative to the LPS group alone (Fig. 7C). Hierarchical clustering heatmaps demonstrated that LPS stimulation markedly upregulated the expression of genes participating in pro-inflammation, oxidative stress, and ferroptosis (e.g., *Il1b*, *Acsl4*, *Txnip*, *Lpcat3*, *Crem*, *Csf2rb*), an effect significantly reversed by Cu_4_ cluster treatment. Conversely, Cu_4_ clusters promoted anti-inflammatory, antioxidant, and anti-ferroptosis genes (e.g., *Hmox1*, *Gstp3*, *Slc40a1*, *Cybb*, *Ftl*) to be highly expressed (Fig. 7D). To further explore the potential biological processes influenced by Cu_4_ clusters, we conducted a GO enrichment analysis on DEGs between the LPS and LPS + Cu_4_ groups. As shown in Fig. 7E, processes well contributing to the therapeutic efficacy of Cu_4_ clusters included innate immune response regulation, TNF production regulation, NF-κB signal transduction, OC differentiation, and negative regulation of cytokine production. KEGG pathway enrichment analysis (Fig. 7F) revealed significant enrichment in pathways including Toll-like receptor signaling, MAPK signaling, p53 signaling, OC differentiation, NF-κB signaling, JAK-STAT signaling, and ferroptosis, suggesting their potential involvement in the Cu_4_ cluster therapeutic mechanism. Furthermore, gene set enrichment analysis (GSEA) demonstrated obvious enrichment and upregulation of oxidative phosphorylation-related genes following Cu_4_ cluster treatment compared to the LPS group (Fig. 7G). Collectively, Cu_4_ clusters may demonstrate their anti-inflammatory impact through scavenging ROS and upregulating endogenous anti-inflammatory and antioxidant responses, thereby attenuating inflammation, oxidative stress, and ferroptosis, promoting cellular functions exemplified by oxidative phosphorylation to be recovered.

### Activation of Nrf2-mediated antioxidant pathway and regulation of ferroptosis by Cu₄ clusters

**Fig. 8:**
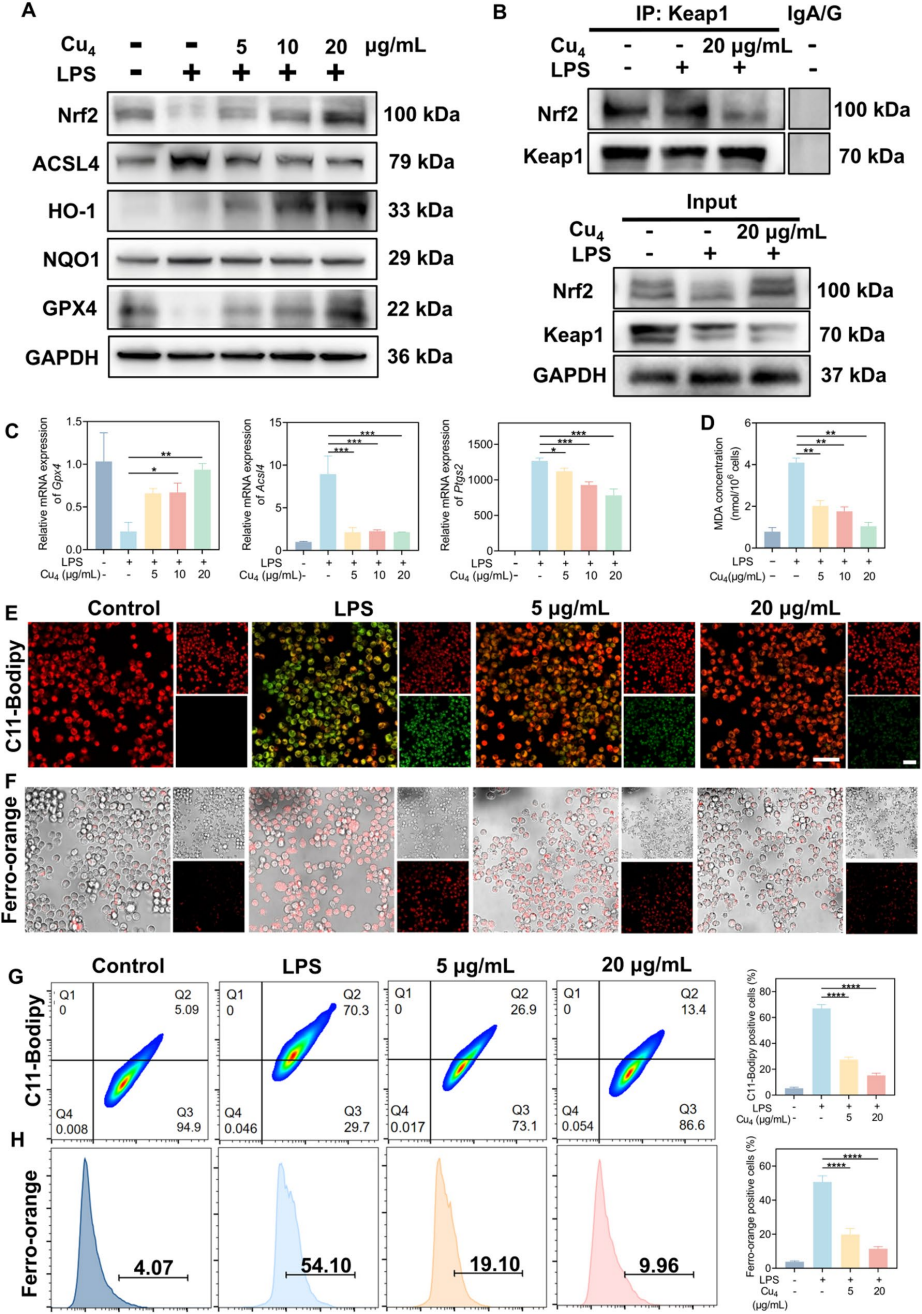
Activation of Nrf2-mediated antioxidant pathway and regulation of ferroptosis by Cu₄ clusters. (**A**) WB analysis of Nrf2, HO-1, NQO1, ACSL4, and GPX4 with various treatment groups. (**B**) Co-immunoprecipitation assays of Keap1-Nrf2. (**C**) RT-qPCR analysis of ferroptosis-related gene expression in LPS-activated RAW 264.7 macrophages. (**D**) Intracellular MDA levels in RAW 264.7 macrophages. (**E**, **F**) Confocal images, and (**G**, **H**) FCM analysis of RAW 264.7 macrophages after C11-Bodipy and Ferro-orange staining. Data are in the format of the mean ± SD, *n* = 3, Scale bar = 50 μm, *p < 0.05; **p < 0.01; ***p < 0.001

Existing literature has extensively explored as well as validated Nrf2 pathway that targets ROS-scavenging and anti-inflammatory activities [43, 44]. This study also aimed at evaluating the regulatory mechanism of Cu₄ clusters against the Nrf2-related pathway, a key pathway against oxidative stress and inflammation [38]. According to RNA sequencing analysis, Cu_4_ cluster treatment promoted Nrf2-downstream genes possessing anti-inflammatory and anti-oxidative features (*Hmox1*, *Nqo1*) to be largely expressed, hence, we performed Western blotting analysis to validate these results (Fig. 8A, S17). LPS stimulation induced a slight induction of Nrf2, HO-1, and NQO1 expression, yet this was insufficient to fully counteract oxidative stress, conforming to the RNA-seq findings. In contrast, Cu_4_ clusters dose-dependently upregulated Nrf2 (a master regulator of antioxidant responses) as well as its downstream effectors HO-1 (a key anti-oxidative enzyme) and NQO1 (a cytoprotective protein) [45]. This activation at the protein level directly corroborated the transcriptional upregulation of Nrf2 pathway-related genes (such as *Hmox1*) observed in the RNA-seq data. To elucidate the underlying mechanism, we conducted Co-immunoprecipitation assays of Keap1-Nrf2 to investigate whether the Cu_4_ clusters would affect the interaction between Keap1 and Nrf2 in LPS-activated macrophages. The results confirmed that after treatment with the Cu_4_ clusters, the immunoprecipitation level of Keap1 and Nrf2 was lower (Fig. 8B). By boosting the endogenous antioxidant system, the clusters complement their exogenous ROS-scavenging activity to form a dual-layer antioxidant defense, which is more effective at breaking the “oxidative stress-inflammation” cycle.

According to RNA-seq results, LPS treatment induced an increase in ferroptosis-related genes in RAW 264.7 macrophages, while Cu₄ treatment can enhance the expression of anti-ferroptosis genes. Thus, we utilized multiple approaches to test the regulatory role of Cu₄ clusters in cellular ferroptosis. During ferroptosis, ACSL4 is widely recognized as a key promoter, as it facilitates the integration of polyunsaturated fatty acids of arachidonic acid (AA) into phospholipids, thereby providing substrates for lipid peroxidation [46]. According to Fig. 8A and S17, LPS treatment remarkably upregulated intracellular ACSL4 expression, while Cu₄ treatment effectively suppressed this upregulation. Conversely, GPX4 plays a central role in maintaining intracellular lipid peroxidation balance and is regarded as a critical inhibitor of ferroptosis [47, 48]. The results of Western blotting demonstrated that Cu₄ treatment effectively increased GPX4 expression, collectively confirming that Cu₄ clusters can counteract cellular ferroptosis by regulating these key mediators. The dual regulation of ACSL4 and GPX4 by Cu₄ clusters is mechanistically significant: ACSL4 promotes ferroptosis by generating lipid peroxidation substrates, while GPX4 inhibits ferroptosis by reducing lipid peroxides. By simultaneously downregulating ACSL4 (cutting off substrate supply) and upregulating GPX4 (enhancing peroxide clearance), Cu₄ clusters target two opposing nodes of the ferroptosis cascade.In addition, these findings were further corroborated by RT-qPCR analysis (Fig. 8C). Transcriptional and translational validation of Nrf2/ferroptosis-related genes strengthened the reliability of the regulatory mechanisms identified.

To further validate the inhibitory effect of Cu₄ clusters on ferroptosis, we assessed lipid peroxidation and iron homeostasis. MDA, a critical biomarker of lipid peroxidation [49], was significantly elevated in LPS-triggered RAW 264.7 macrophages (Fig. 8D), indicating severe oxidative damage to cellular lipids. With the highest concentration (20 µg/mL) treatment of Cu_4_ clusters, MDA restored to near-basal levels. C11-Bodipy assays (Fig. 8E and G, S18) further confirmed lipid peroxidation dynamics: LPS-induced green fluorescence (oxidized lipids) was prominent, while Cu₄ treatment dose-dependently enhanced red fluorescence (unoxidized probe). Ferro-orange staining (Fig. 8F and H, S19) was employed to visualize intracellular labile iron pools, a key driver of ferroptosis via Fenton reactions [50, 51]. LPS stimulation considerably strengthened the red fluorescence intensity (54.1% positive cells), reflecting iron overload, whereas Cu₄ treatment effectively diminished this signal, with 20 µg/mL Cu₄ clusters reducing positive cells to 9.96%, indicative of restored iron homeostasis. The sequencing results indicated that the expression of *Slc40a1* in the LPS group was lower than that in the control group, and Cu_4_ was able to reverse this alteration (Fig. S20). The PCR results corroborate this finding (Fig. S21). *Slc40a1* is the gene responsible for encoding the FPN1 protein. The FPN1 protein facilitates the transport of Fe^2+^ from inside the cell to the exterior. Consequently, Cu_4_ decreases labile Fe²⁺ levels by upregulating *Slc40a1* and enhancing the efflux of Fe²⁺, which is consistent with the results of the Ferro-Orange staining. Collectively, these results demonstrated that Cu₄ inhibits ferroptosis in RAW 264.7 macrophages through dual mechanisms: limiting labile iron accumulation and suppressing lipid peroxidation, thereby disrupting the ferroptosis cascade at multiple critical nodes.

### In vivo inhibitory impact of Cu_4_ clusters on LPS-triggered calvarial osteolysis

**Fig. 9:**
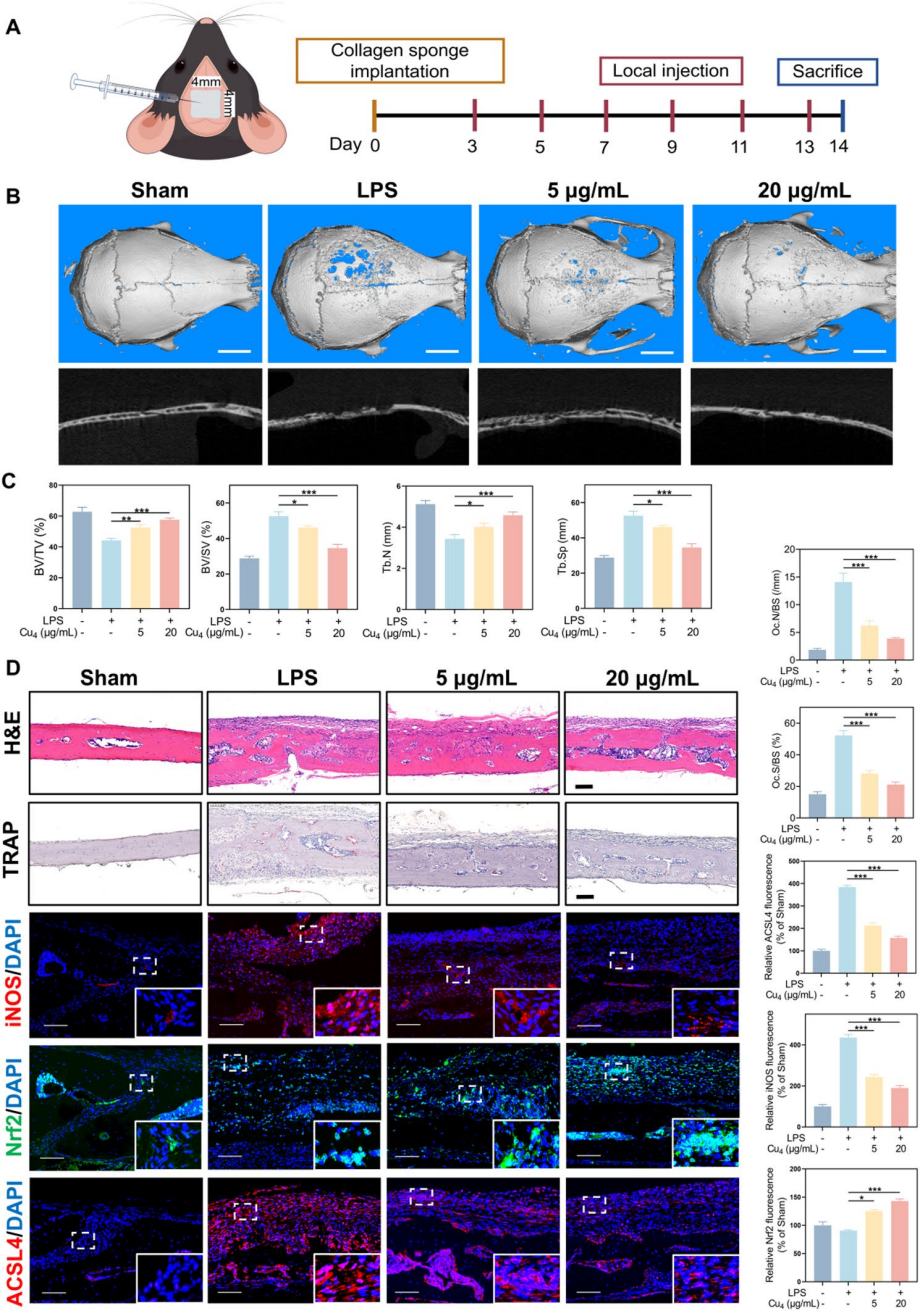
In vivo inhibitory impact of Cu_4_ clusters on LPS-triggered calvarial osteolysis. (**A**) Schematic plot explaining the in vivo experiments. (**B**) Representative micro-CT scanning images of mice calvaria, Scale bar = 2 mm. (**C**) The microstructure indicators of BV/TV, BV/SV, Tb.N, and Tb.Sp were measured and analyzed. (**D**) Representative H&E, TRAP, and immunostaining images of iNOS, Nrf2, and ACSL4, Scale bar = 100 μm. Data are in the format of the mean ± SD, *n* = 3, *p < 0.05; **p < 0.01; ***p < 0.001

After confirming the in vitro biocompatibility, cell internalization, anti-inflammatory characteristics, and fundamental anti-inflammatory pathways of Cu_4_, we assessed their in vivo therapeutic effectiveness by creating a murine model with regard to LPS-induced calvarial osteolysis. Fig. 9A illustrates the surgical implantation of LPS-impregnating collagen sponge blocks into mice’ calvaria to trigger inflammation-mediated bone resorption [52]. Subsequently, different concentrations of normal saline or Cu_4_ were injected locally every two days. 14 days of treatment later, samples of the mouse skulls were obtained for radiological and histological evaluations.

According to micro-CT in Fig. 9B, the Sham group exhibits an intact calvarial structure. In the LPS group, severe osteolysis occurred, with obvious bone defects. In contrast, the Cu_4_ clusters-treated groups exhibited a significant reduction in osteolytic lesions. The higher the concentration of Cu_4_, the more intact the calvarial structure tended to be. Quantitative analysis further disclosed that the Cu_4_ cluster-treated group presented obviously enhanced critical morphometric parameters including bone volume/tissue volume (BV/TV), bone volume/spacer volume (BV/SV), trabecular bone number (Tb.N), and trabecular bone separation (Tb.Sp) versus the LPS-induced group (Fig. 9C). This set of parameters collectively reflects the positive impact of Cu_4_ clusters on bone microarchitecture. The improvement in these parameters in the Cu_4_ group underscores its efficacy in alleviating LPS-induced calvarial osteolysis at the structural level. The dose-dependent recovery of bone microarchitecture parameters is clinically meaningful: BV/TV and BV/SV directly reflect bone mass, Tb.N indicates trabecular density, and Tb.Sp reflects trabecular connectivity - all are key indicators of bone strength and prosthesis stability in clinical practice. The restoration of these parameters confirms that Cu₄ clusters actively protect bone structural integrity, which is essential for preventing prosthesis loosening (the ultimate clinical consequence of inflammatory osteolysis).

Histological analysis (Fig. 9D) further supported these findings. According to H&E staining, the Sham group had a normal calvarial tissue structure. The LPS group was accompanied by significant tissue damage and inflammatory cell infiltration. Conversely, in the Cu_4_-treated groups, the tissue structure was comparatively well-maintained, with increasingly less inflammatory cells, particularly in the 20 µg/mL Cu_4_ clusters group. TRAP staining engaged in identifying OCs, revealed a substantial amount of TRAP-positive OCs in the LPS group, while the Cu_4_ clusters-treated groups had fewer TRAP-positive cells, indicating that Cu_4_ could inhibit OC formation *in vivo.*

Immunofluorescence staining provided insights into the molecular mechanisms. The iNOS (a marker of M1-type pro-inflammatory macrophages) expression was significantly down-regulated in the Cu_4_-treated groups, suggesting that Cu_4_ suppressed macrophage M1 polarization. For Nrf2, its nuclear translocation was promoted in the Cu_4_-treated groups versus the LPS group, which was consistent with the in vitro findings that Cu_4_ activated the Nrf2 pathway to exert anti-inflammatory effects. Additionally, ACSL4, a marker related to ferroptosis, was highly expressed in the LPS group, while Cu_4_ treatment reduced its expression, indicating that Cu_4_ could also regulate ferroptosis in vivo to mitigate inflammatory osteolysis, which was in line with the earlier proposed mechanism of action. Besides, major organs (heart, liver, spleen, lung, and kidney) underwent hematological analyses (Table S2) and H&E staining following treatment (Fig. S22). The results demonstrated that, upon completion of the 14-day treatment cycle, Cu_4_ clusters did not induce apparent organ damage in the mice, verifying their satisfactory biosafety profile. The biodistribution of the Cu_4_ clusters, as quantified by ICP-MS, demonstrated predominant accumulation in the kidneys at 48 h post-injection (Fig. S23). This distribution profile aligns with previously reported behavior of ultrasmall metal clusters, and suggests a primary renal clearance pathway for Cu_4_ clusters [16, 17].

## Conclusion

In conclusion, we successfully designed ultrasmall Cu₄ clusters. By taking ROS - the core driver of oxidative stress - as the hub, systematically illustrating the multi-effect synergistic mechanism of Cu₄ clusters in intervening inflammatory osteolysis, which follows the cascade of “ROS scavenging → dual-pathway regulation of Nrf2/NF-κB → ferroptosis inhibition → osteoclast suppression”. Compared with existing nanozymes or bone repair strategies that focus on a single target, this study reveals the multi-dimensional intervention mode of metal nanoclusters in inflammatory osteolysis, encompassing “antioxidation-anti-inflammation-ferroptosis regulation-osteoclast inhibition”. Its innovation lies in identifying ROS metabolic disorder as a key node integrating “inflammatory microenvironment - cell death - abnormal bone metabolism”, enabling the synergistic regulation of multiple pathways through a single nanomaterial. From a clinical translation perspective, Cu₄ clusters not only serve directly as therapeutic agents for inflammatory osteolysis, but can also be applied as surface coatings for orthopedic implants such as artificial joints. Collectively, Cu₄ clusters contribute to a novel and promising therapeutic approach that could potentially revolutionize the treatment of inflammatory osteolysis, while also holding significant translational value for addressing a broader spectrum of inflammation-associated disorders, thereby assisting in the development of multi-mechanistic therapies in the field of inflammatory disease management from new perspectives.

## Supplementary Information

Supplementary Material 1

## Data Availability

No datasets were generated or analysed during the current study.

## Abbreviations

ANOVA: One-way analysis of variance
BCA: Bicinchoninic acid
BMMs: Bone marrow macrophages
CAT: Catalase
CCK-8: Cell Counting Kit-8
CLSM: Confocal laser scanning microscopy
CSF: Macrophage colony-stimulating factor
DCFH-DA: Dichloro-dihydro-fluorescein diacetate
DEGs: Differentially expressed genes
DHE: Dihydroethidium
FBS: Fetal bovine serum
GO: Gene ontology
HRP: Horseradish peroxidase
IL: Interleukin
KEGG: Kyoto Encyclopedia of Genes and Genomes
LPS: Lipopolysaccharide
MDA: Malondialdehyde
α-MEM: Minimal essential medium alpha
MMP: Membrane potential
MMP9: Matrix metalloproteinase 9
NF-κB: Nuclear factor kappa-B
iNOS: Inducible nitric oxide synthase
Nrf2: Nuclear factor erythroid 2-related factor
NSAIDs: Non-steroidal anti-inflammatory drugs
O_2_^−^: Superoxide radicals
OC: Osteoclast
PBS: Phosphate buffered saline
PCR: Polymerase chain reaction
PFA: Paraformaldehyde
PI: Propidium iodide
PVDF: Polyvinylidene fluoride
RANKL: Receptor activator of NF-κB ligand
RBC: Red blood cell
ROS: Reactive oxygen species
mtROS: Mitochondrial ROS
SOD: Superoxide dismutase
TEM: Transmission electron microscopy
TJR: Total joint replacement
TRAP: Tartrate-resistant acid phosphatase
TNF: Tumor necrosis factor
