# Supplementary material for: Clusterzymes-driven therapy: ultrasmall Cu4 nanoclusters achieve dual-pronged synergistic effects on antioxidant defense and ferroptosis Inhibition for inflammatory osteolysis
Source: J Nanobiotechnology. 2026 Jan 21;24:91. doi: 10.1186/s12951-025-04009-2 (PMC12853966; doi:10.1186/s12951-025-04009-2)
Supplement: Supplementary file 1 — Supplementary Material 1 [file 12951_2025_4009_MOESM1_ESM.docx]

**Supporting Information**

Clusterzymes-Driven Therapy: Ultrasmall Cu_4_ Nanoclusters Achieve Dual-Pronged Synergistic Effects on Antioxidant Defense and Ferroptosis Inhibition for Inflammatory Osteolysis

*Yucheng Wang^a,b,1^, Zeyu Han^c,1,^, Guanghua Hao^d,1^, Han Cheng^e^, Fengrong Dai^f,*^, Xuzhuo Chen^c,*^, Bin Shi^a,b,*^*

^a^Department of Oral and Maxillofacial Surgery, the First Affiliated Hospital of Fujian Medical University, Fuzhou, 350005, China.

^b^School of Stomatology, Fujian Medical University, Fuzhou, 350005, China

^c^Department of Oral Surgery, Shanghai Ninth People's Hospital, Shanghai Jiao Tong University School of Medicine; College of Stomatology, Shanghai Jiao Tong University; National Center for Stomatology; National Clinical Research Center for Oral Diseases; Shanghai Key Laboratory of Stomatology; Shanghai Research Institute of Stomatology, Shanghai, 200011, China

^d^Department of operating room，Linyi Central Hospital, Linyi 276400, China

^e^School of Physical Science and Technology, Shanghai Tech University, Shanghai 201210, China

^f^State Key Laboratory of Structural Chemistry, Fujian Institute of Research on the Structure of Matter, Chinese Academy of Sciences, Fuzhou, Fujian

^*^Corresponding authors: Fengrong Dai, Fujian Institute of Research on the Structure of Matter, Chinese Academy of Sciences, Fuzhou, 350005, China.

Xuzhuo Chen, the Ninth People's Hospital Affiliated to Shanghai Jiao Tong University, 639 Zhizaoju Road, Shanghai, 200011, China. E-mail: cxzzxcaa@126.com

Professor Bin Shi, the First Affiliated Hospital of Fujian Medical University, 20 Chazhong Road, Fuzhou, 350005, China. E-mail: [shibin@fjmu.edu.cn](mailto:Zhang);shibin@fjmu.edu.cn（B)

^1^These authors contributed equally.


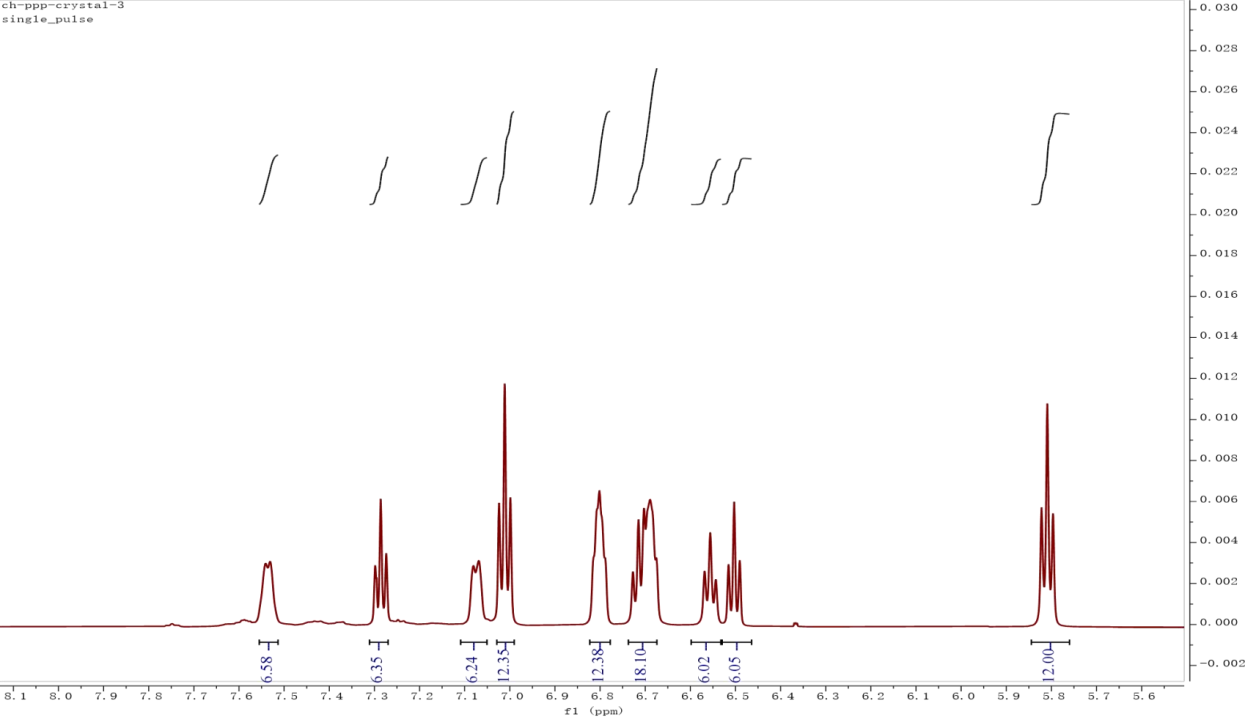


**Figure S1.** The ^1^H NMR spectrum of Cu_4_ clusters in CD_2_Cl_2_ at room temperature.


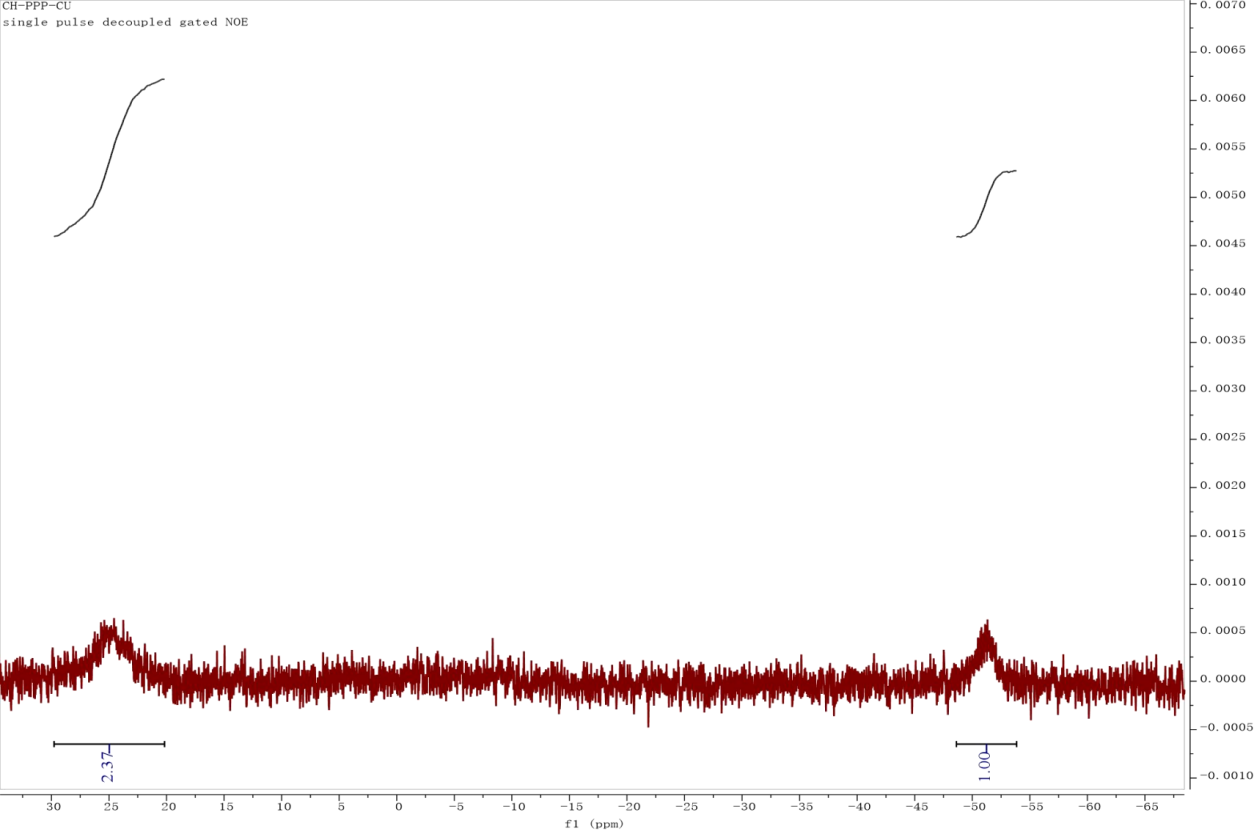


**Figure S2.** The ^31^P NMR spectrum of Cu_4_ clusters in CD_2_Cl_2_ at ambient temperature.


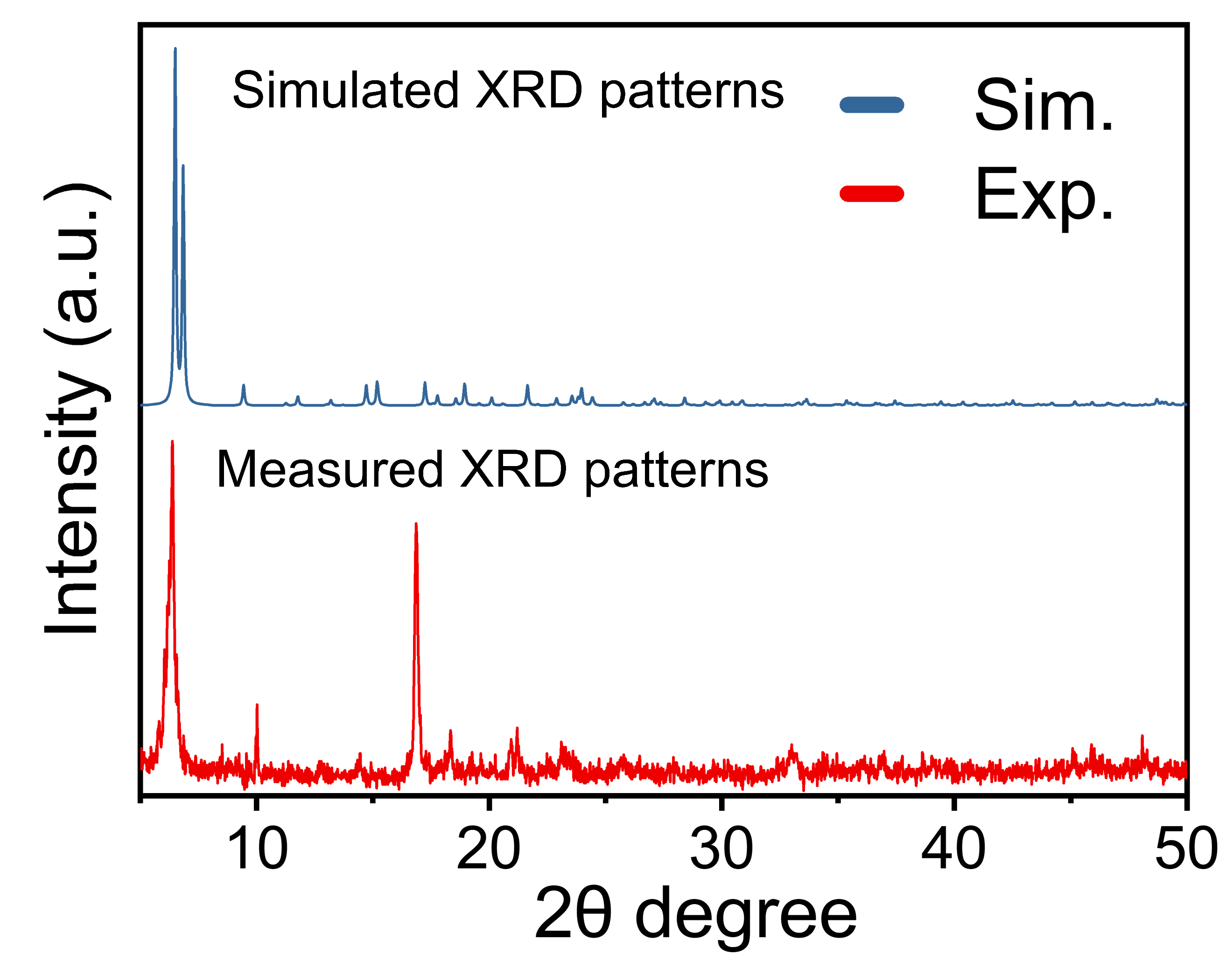


**Figure S3.** PXRD patterns of Cu_4_ clusters.


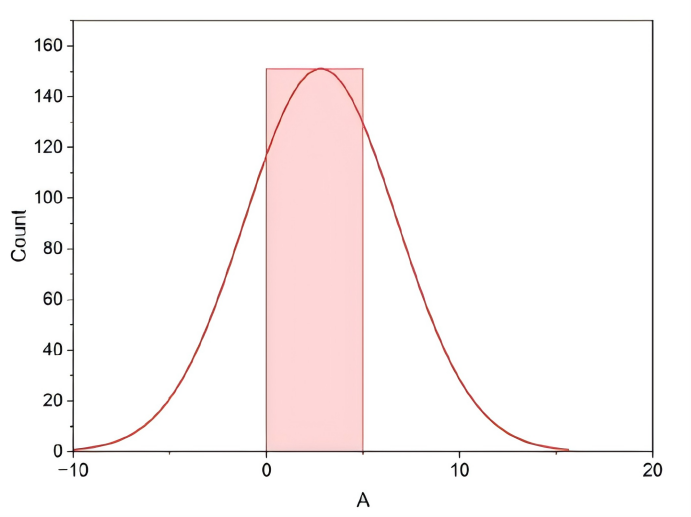


**Figure S4**. Size distribution histogram of Cu_4_ cluster.


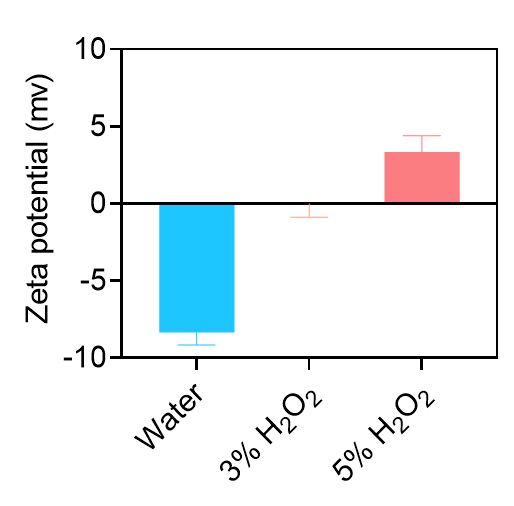


**Figure S5.** Zeta potential of Cu_4_ clusters before and after ROS treatment.


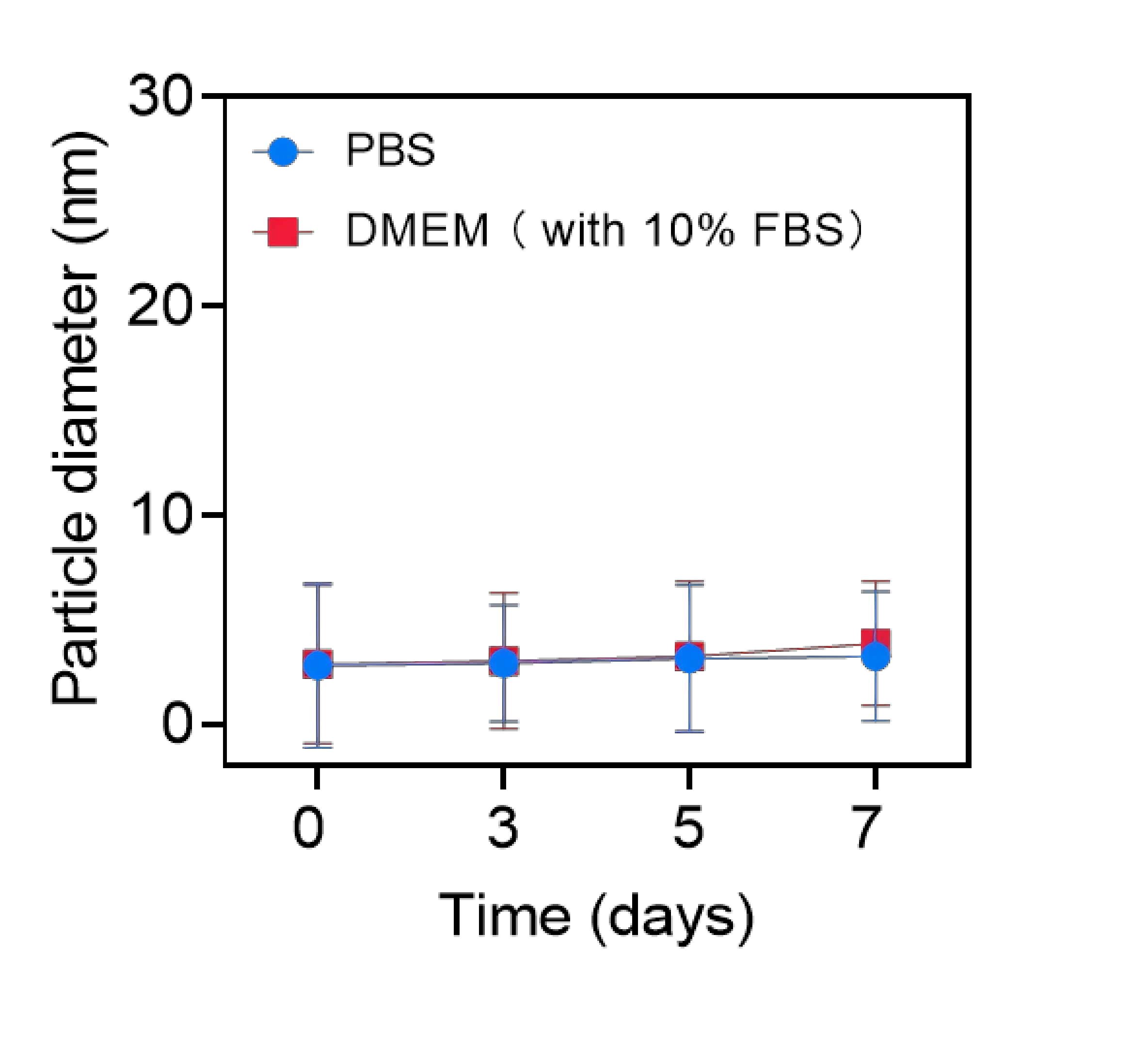


**Figure S6**. Cu_4_ clusters stability in biological media (FBS, PBS) over time.


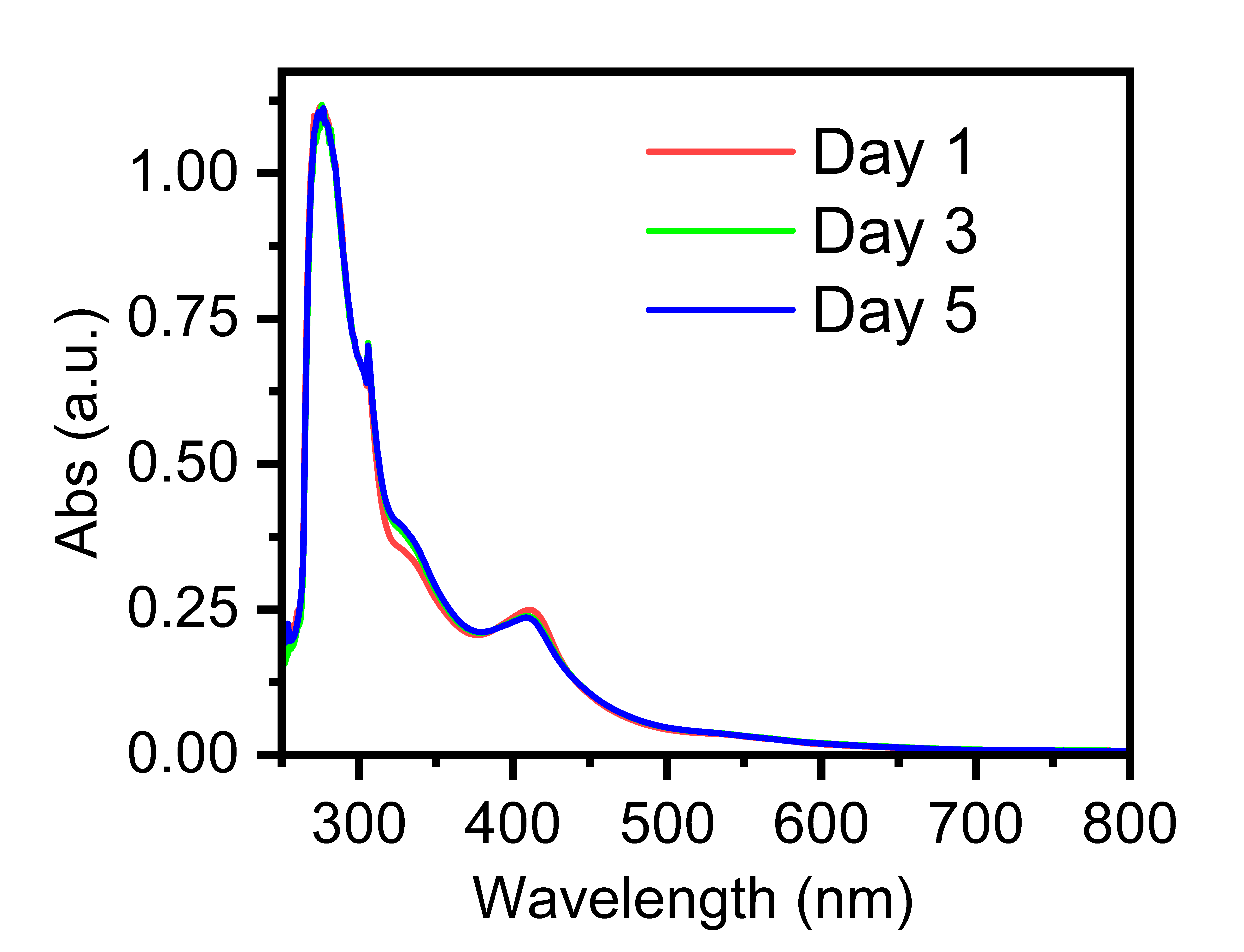


**Figure S7**. UV-Vis absorption spectra of Cu_4_ clusters in serum, recorded at different duration (1 to 5 days).


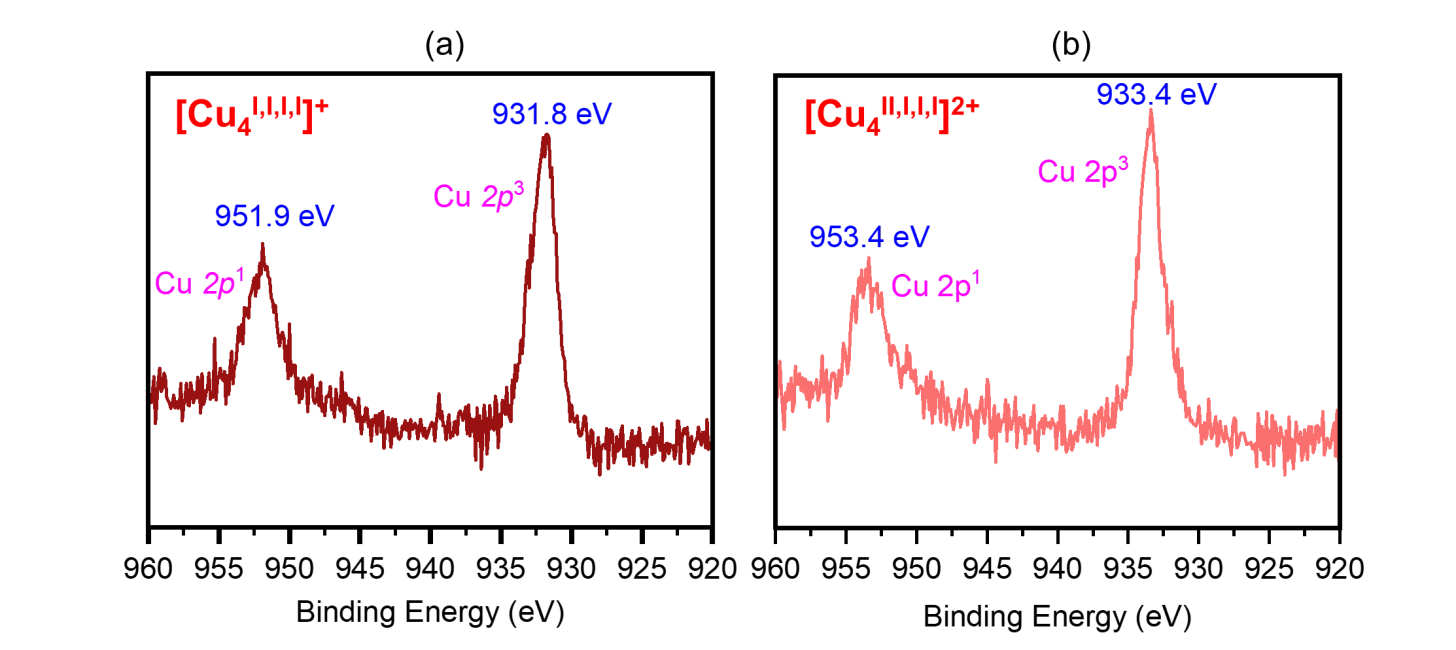


**Figure S8.** The XPS spectra of Cu_4_I,I,I,I cluster (A) and its one-electron oxidized Cu_4_II,I,I,I cluster with mixed-valence (B).


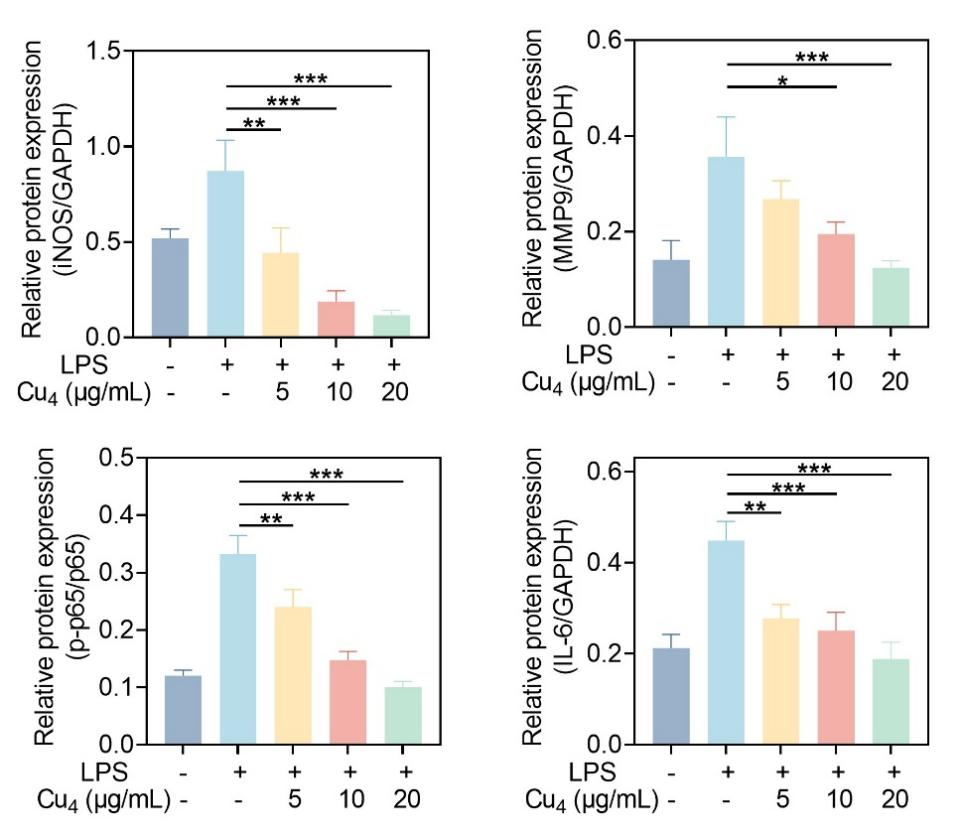


**Figure S9.** Quantitative analysis of protein expression of iNOS, MMP9, p-p65, p65, and IL-6. Data are in the format of the mean ± SD, n= 3, **p*< 0.05; ***p*< 0.01; ****p*< 0.001.


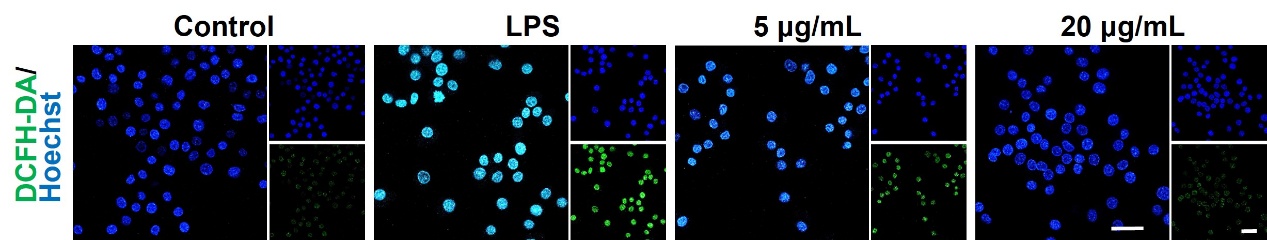


**Figure S10.** Confocal images of RAW 264.7 macrophages treated with various concentrations of Cu_4_ clusters and stained with DCFH-DA, Scale bar = 50 μm.


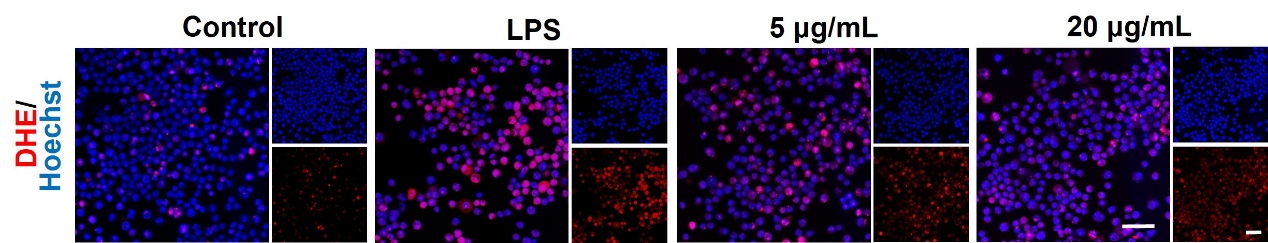


**Figure S11.** Confocal images of RAW 264.7 macrophages treated with various concentrations of Cu_4_ clusters and stained with DHE, Scale bar = 50 μm.

**
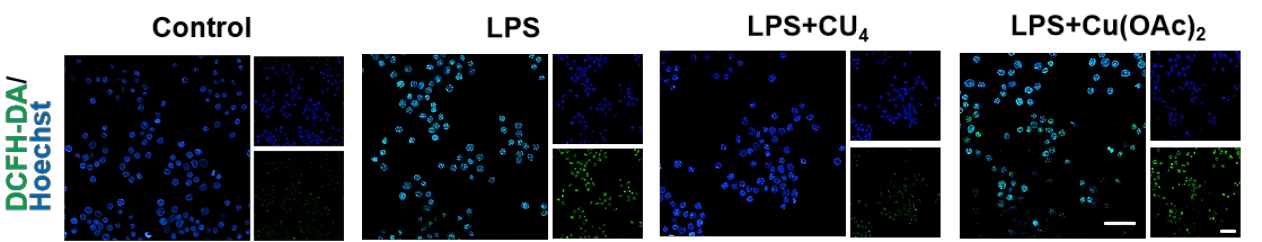
**

**Figure S12**. Confocal images of RAW 264.7 macrophages treated with Cu_4_ clusters or Cu(OAc)_2_ and stained with DCFH-DA, Scale bar = 50 μm.


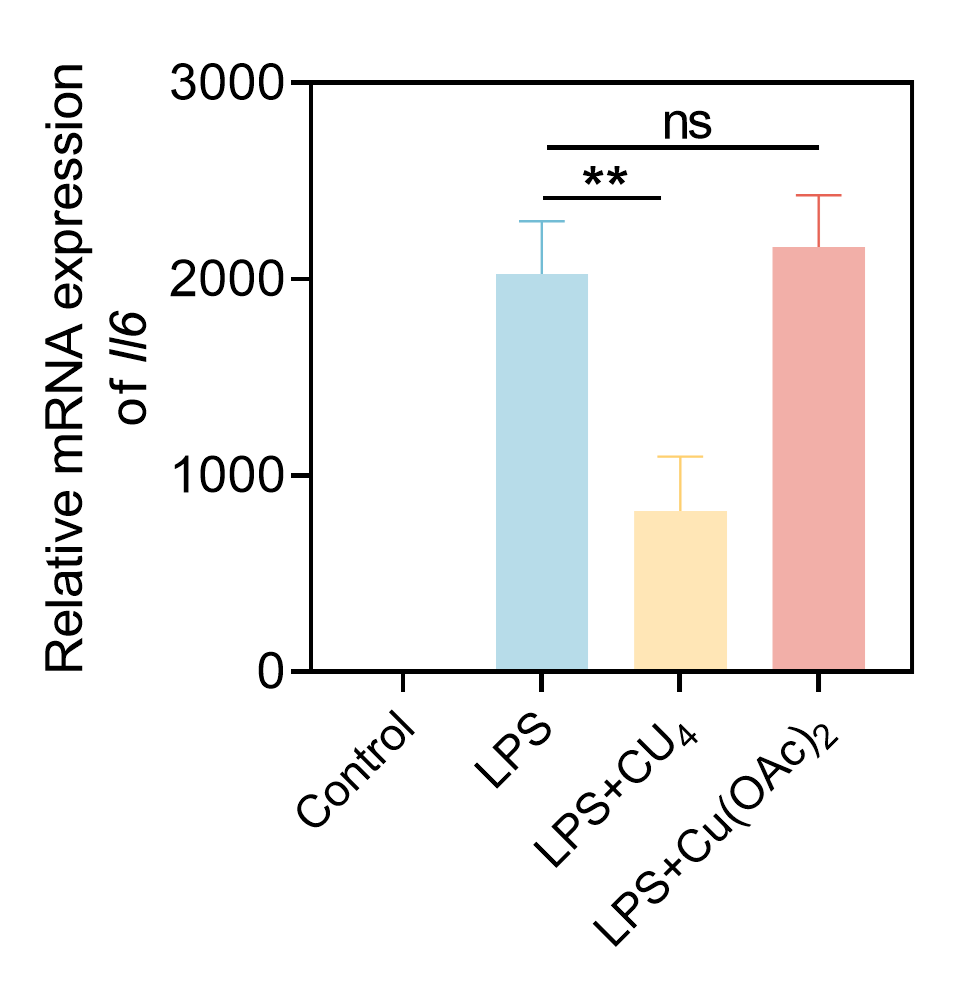

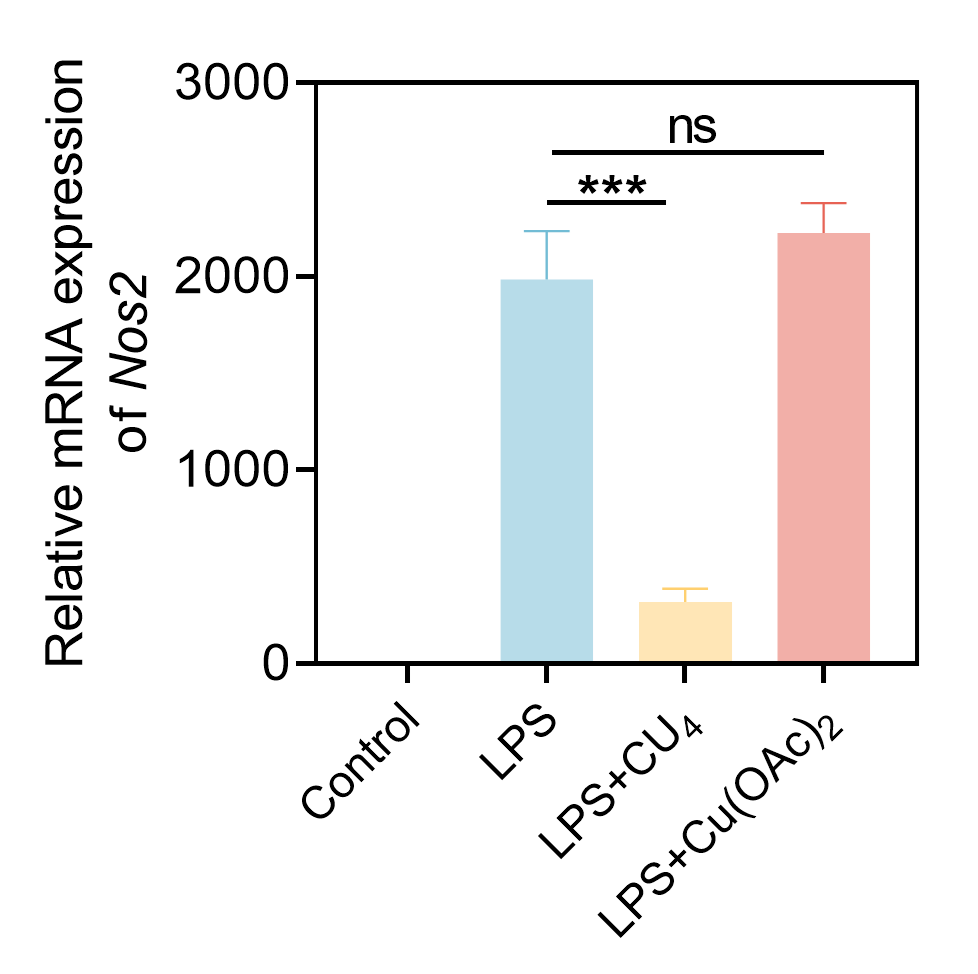


**Figure S13**. RT-qPCR analysis of pro-inflammatory gene expression in RAW 264.7 macrophages after Cu_4_ clusters or Cu(OAc)_2_ treatment. Data are in the format of the mean ± SD, n= 3, **p*< 0.05; ***p*< 0.01; ****p*< 0.001.

**.**

**
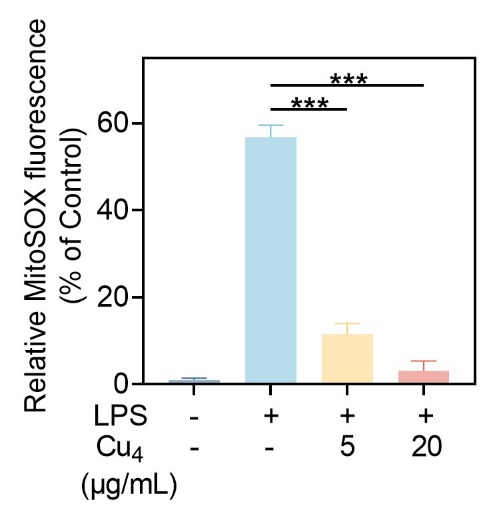
**

**Figure S14.** Quantitative analysis of fluorescence of Mitosox. Data are in the format of the mean ± SD, n= 3, **p*< 0.05; ***p*< 0.01; ****p*< 0.001.


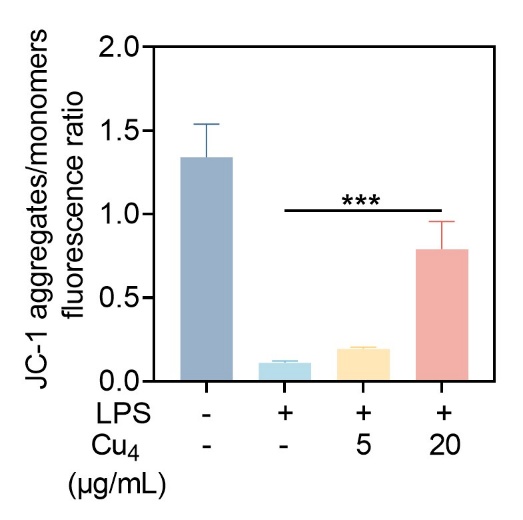


**Figure S15.** Quantitative analysis of fluorescence of JC-1. Data are in the format of the mean ± SD, n= 3, **p*< 0.05; ***p*< 0.01; ****p*< 0.001.


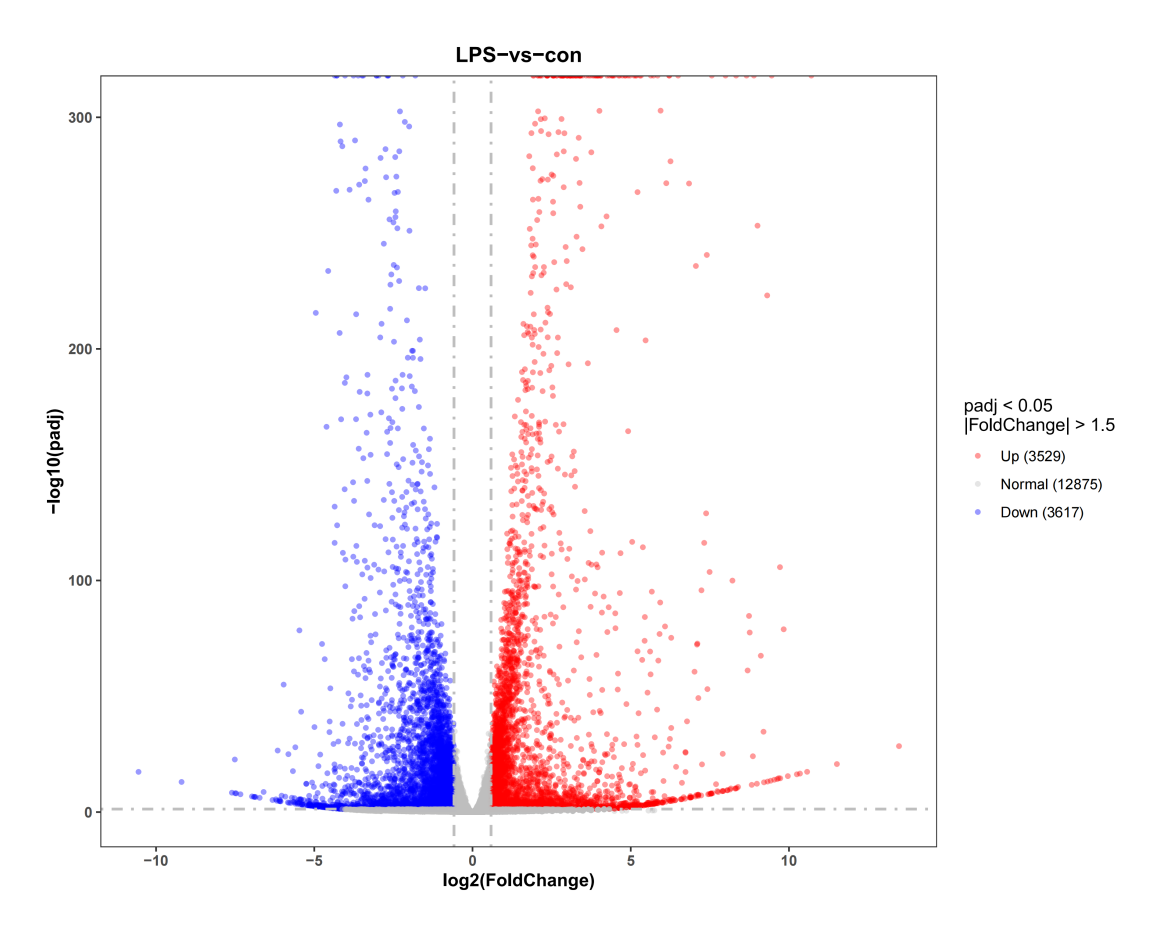


**Figure S16**. Volcano plots illustrating the DEGs between the Con and LPS groups.


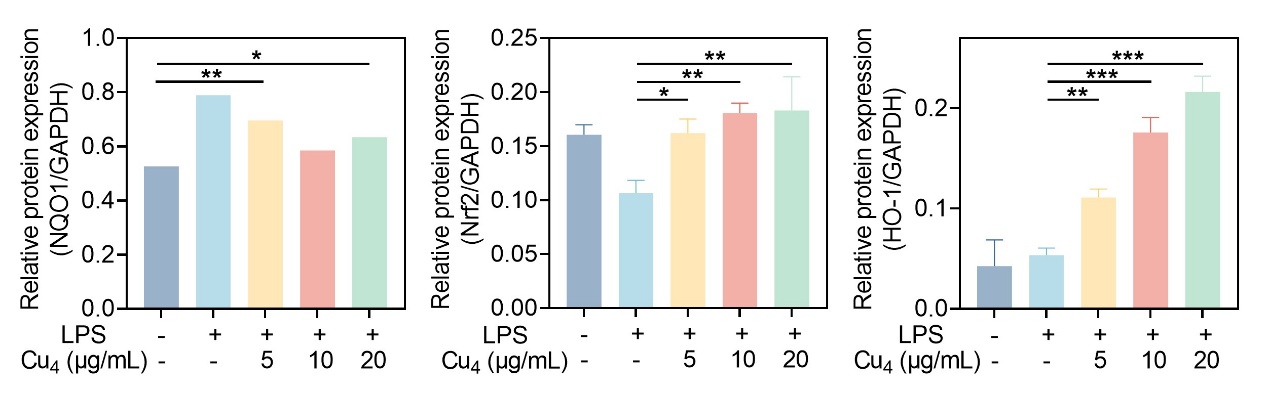


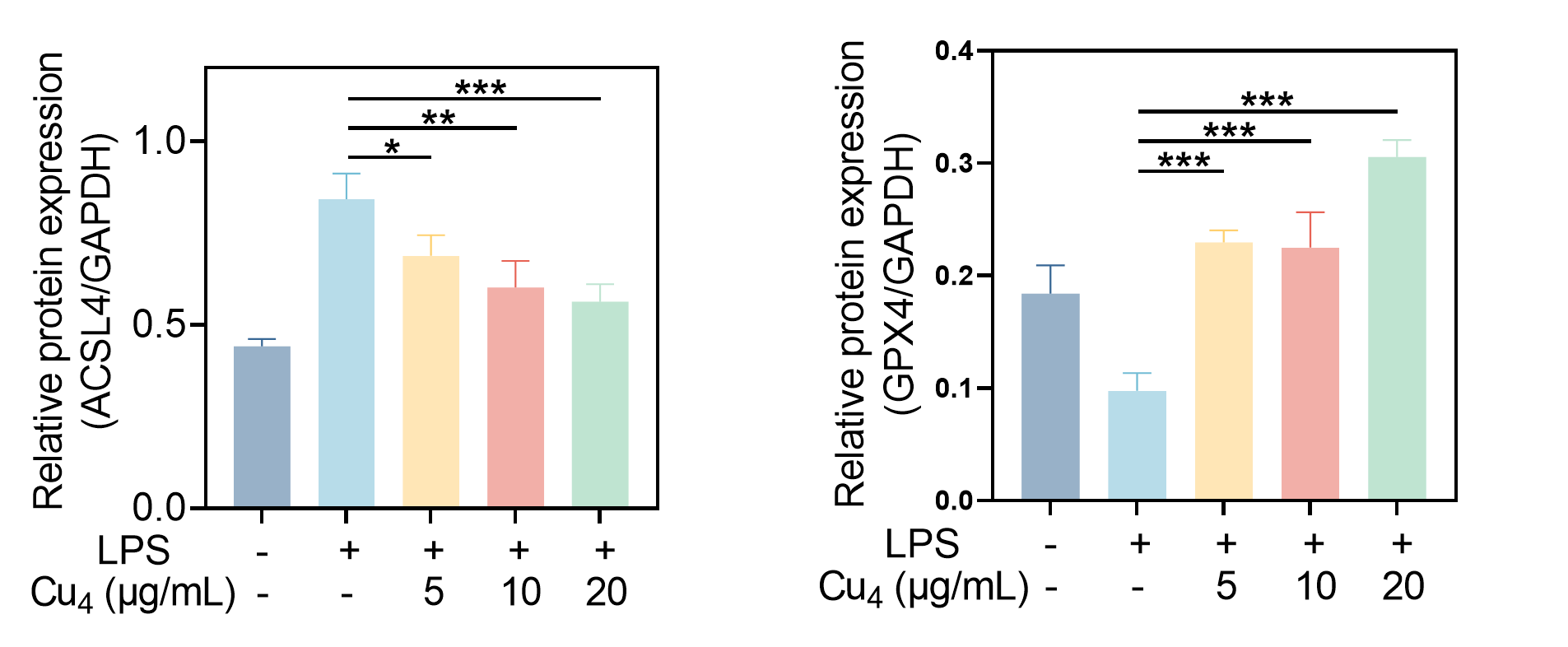


**Figure S17.** Quantitative analysis of protein expression of Nrf2, HO-1, NQO1, ACSL4 and GPX4. Data are in the format of the mean ± SD, n= 3, **p*< 0.05; ***p*< 0.01; ****p*< 0.001.


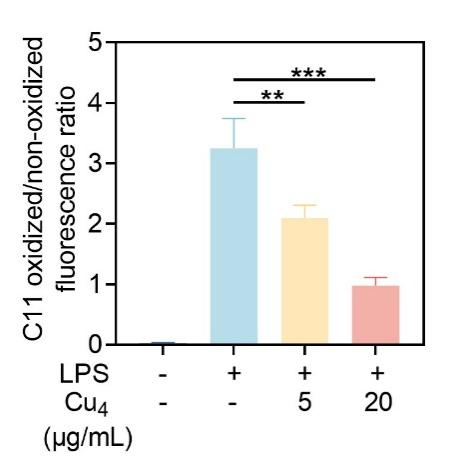


**Figure S18.** Quantitative analysis of fluorescence of C11-Bodipy. Data represent the mean ± SD, n= 3, **p*< 0.05; ***p*< 0.01; ****p*< 0.001.


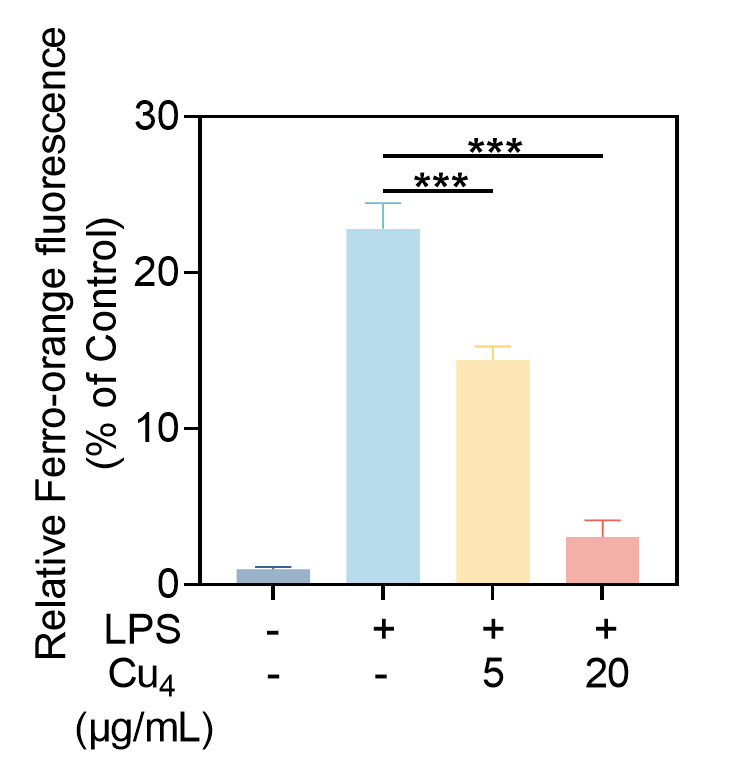


**Figure S19.** Quantitative analysis of fluorescence of Ferro-orange. Data represent the mean ± SD, n= 3, **p*< 0.05; ***p*< 0.01; ****p*< 0.001.


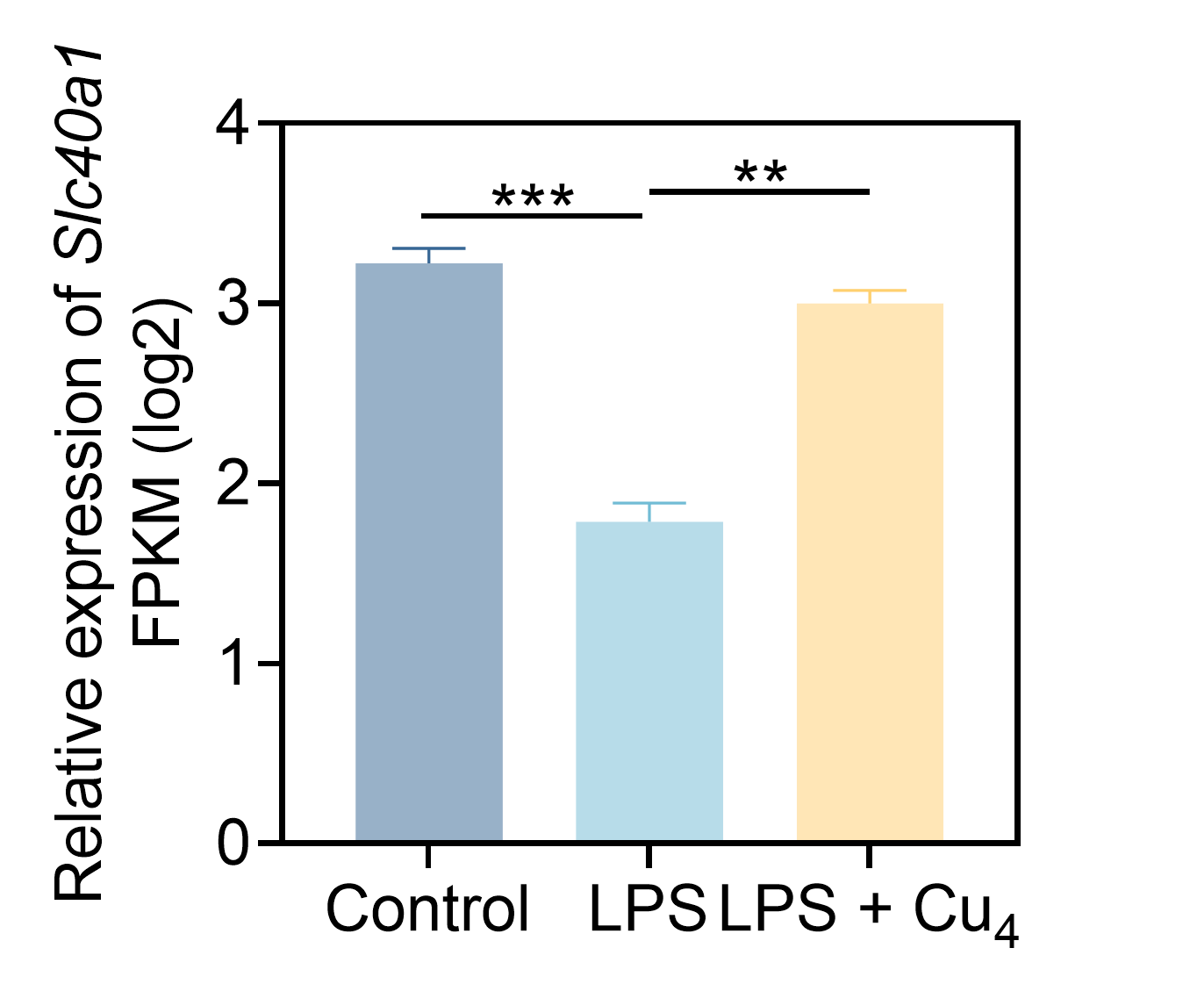


**Figure S20**. Relative expression of *Slc40a1* in RNA-seq. Data are in the format of the mean ± SD, n= 3, **p*< 0.05; ***p*< 0.01; ****p*< 0.001.


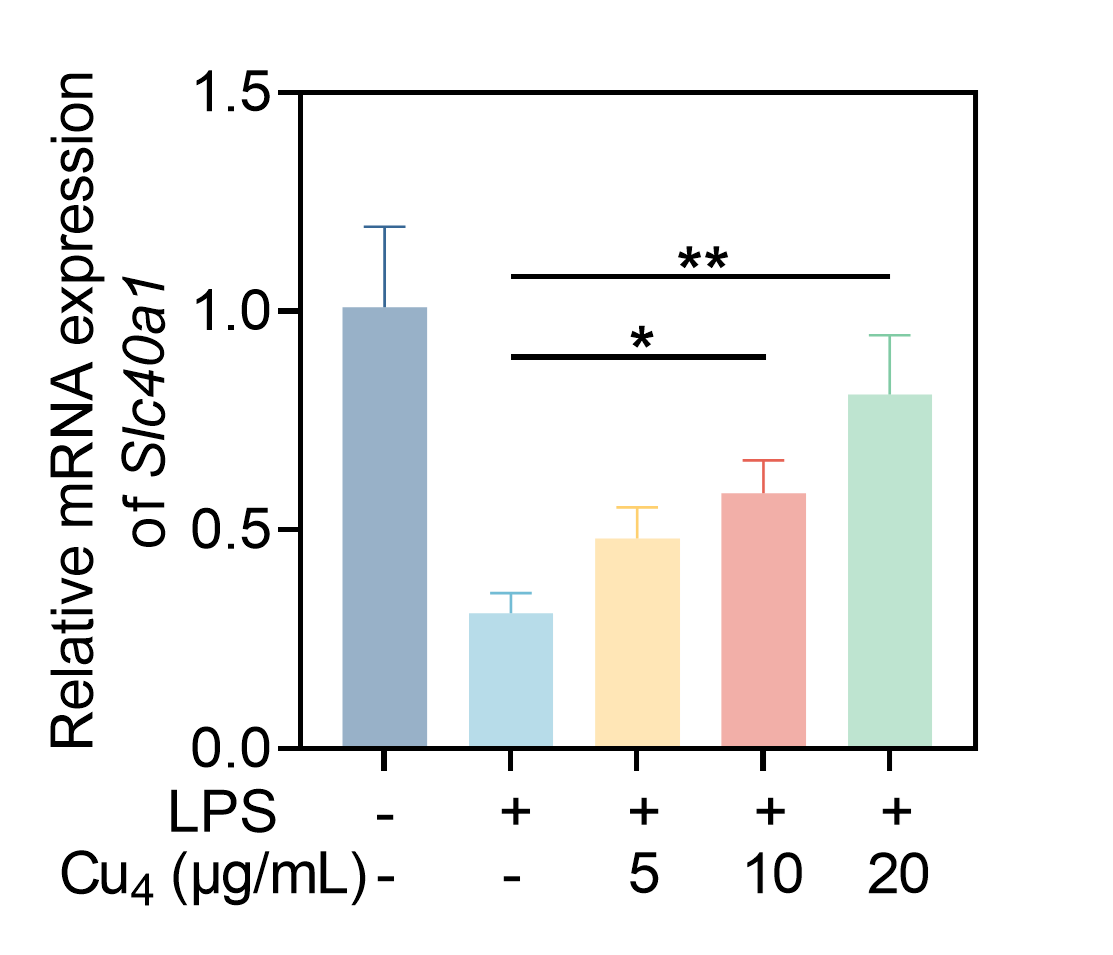


**Figure S21**. RT-qPCR analysis of *Slc40a1* expression in LPS-activated RAW 264.7 macrophages. Data are in the format of the mean ± SD, n= 3, **p*< 0.05; ***p*< 0.01; ****p*< 0.001.


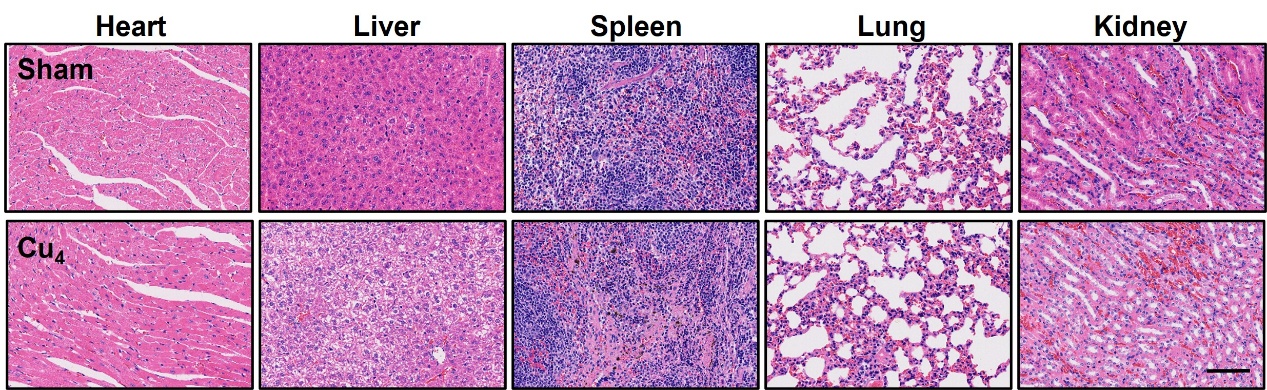


**Figure S22.** H&E staining images of heart, liver, spleen, lung, and kidney 14 days after model establishment (11 days after Cu_4_ clusters treatment), Scale bar = 100 μm.

**
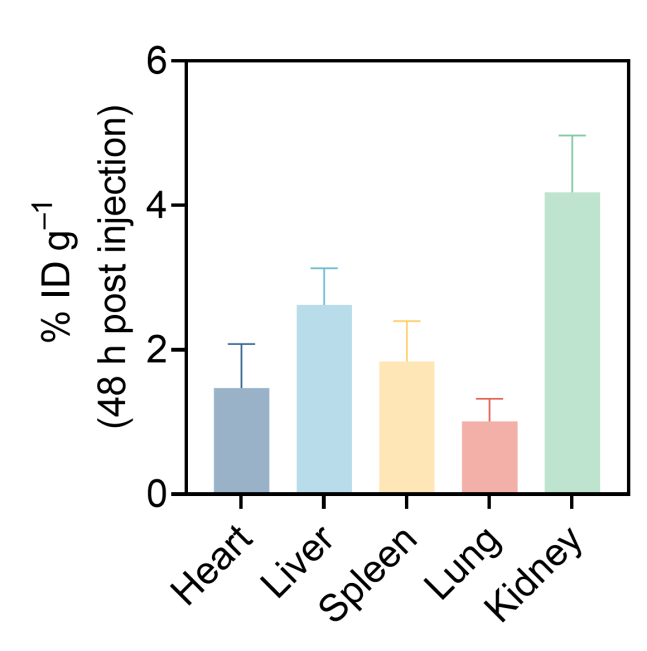
**

**Figure S23**. Biodistribution of Cu_4_ clusters in major organs at 48 h post injection. Data represent the mean ± SD of three independent animals. Data are in the format of the mean ± SD, n= 3.

**Table S1.** List of primer sequences of RT-qPCR

| Gene | Forward | Reverse |
| --- | --- | --- |
| *Gapdh* | ACCCAGAAGACTGTGGATGG | CACATTGGGGGTAGGAACAC |
| *Il1b* | TCGCAGCAGCACATCAACAAGAG | AGGTCCACGGGAAAGACACAGG |
| *Il6* | CTTCTTGGGACTGATGCTGGTGAC | AGGTCTGTTGGGAGTGGTATCCTC |
| *Nos2* | ACTCAGCCAAGCCCTCACCTAC | TCCAATCTCTGCCTATCCGTCTCG |
| *Gpx4* | GTGTAAATGGGGACGATGCC | ACCACGCAGCCGTTCTTATC |
| *Acsl4* | CTCACCATTATATTGCTGCCTGT | TCTCTTTGCCATAGCGTTTTTCT |
| *Ptgs2* | CAGACAACATAAACTGCGCCTT | GATACACCTCTCCACCAATGACC |
| *Trap* | CAAAGAGATCGCCAGAACCG | GAGACGTTGCCAAGGTGATC |
| *Vatpd2* | AAGCCTTTGTTTGACGCTGT | TTCGATGCCTCTGTGAGATG |
| *Dcstamp* | AAAACCCTTGGGCTGTTCTT | AATCATGGACGACTCCTTGG |
| *Ctsk* | CTTCCAATACGTGCAGCAGA | TCTTCAGGGCTTTCTCGTTC |
| *Nfatc1* | TCGAGTTCGATCAGAGCGG | TGGCTGAAGGAACAGCTGAG |
| *Slc40a1* | ACCAAGGCAAGAGATCAAACC | AGACACTGCAAAGTGCCACAT |

**Table S2.** Routine blood test, liver and kidney functions test of mice after 14 days treatment.

|  | Normal range | Control | Cu_4_ |
| --- | --- | --- | --- |
| Routine blood test | |  |  |
| WBC(10^3^/μL) | 0.80-10.60 | 2.78 ± 0.21 | 2.51 ± 0.18 |
| Lym(10^3^/μL) | 0.60-8.90 | 2.66 ± 0.21 | 2.36 ± 0.18 |
| Neu (10^3^/μL) | 0.23-3.60 | 0.34 ± 0.04 | 0.34 ± 0.02 |
| RBC (10^6^/μL) | 6.50-11.50 | 7.68 ± 0.21 | 7.39 ± 0.28 |
| HGB (g/dL) | 11.0-16.5 | 13.70 ± 0.26 | 13.67 ± 0.49 |
| HCT (%) | 35.0-55.0 | 41.20 ± 1.35 | 39.87 ± 1.10 |
| MCV(fL) | 41.0-55.0 | 54.93 ± 0.38 | 55.23 ± 0.49 |
| MCH (pg) | 13.0-18.0 | 14.17 ± 0.55 | 15.20 ± 0.26 |
| MCHC(g/dL) | 30.0-36.0 | 34.03 ± 1.36 | 36.80 ± 1.85 |
| PLT(10^5^/μL) | 4.0-16.0 | 7.73 ± 0.50 | 7.43 ± 0.21 |
| MPV(fL) | 4.0-6.2 | 6.90 ± 0.17 | 6.87 ± 0.32 |
| PDW(%) | 12.0-17.5 | 13.67 ± 0.21 | 13.83 ± 0.25 |
| Liver Function | |  |  |
| ALT(U/L) | 10.06-96.47 | 58.47 ± 0.25 | 58.84 ± 0.35 |
| AST(U/L) | 36.31-235.48 | 17.99 ± 0.36 | 15.99 ± 0.39 |
| Renal Function | |  |  |
| UREA(mmol/L) | 3.9-12.4 | 7.63 ± 0.08 | 7.65 ± 0.08 |
| CRE (μmol/L) | 10.91-85.09 | 12.10 ± 0.55 | 12.47 ± 0.52 |
